# Supplementary figures and images for: Proteomic landscape of tunneling nanotubes reveals CD9 and CD81 tetraspanins as key regulators
Source: eLife. 2024 Sep 9;13:RP99172. doi: 10.7554/eLife.99172 (PMC11383530; doi:10.7554/eLife.99172)

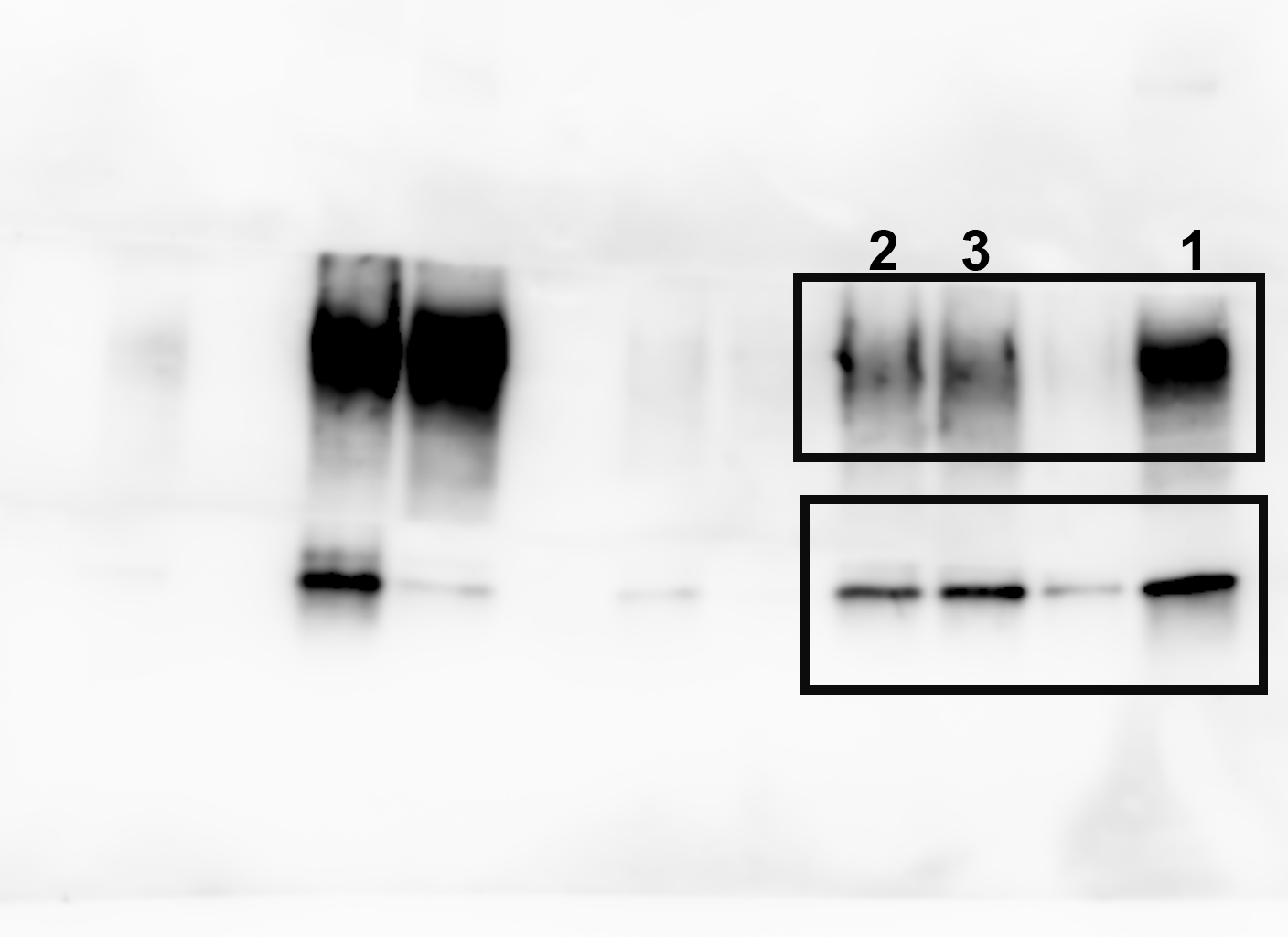

Supplement: Figure 1—source data 1. [file elife-99172-fig1-data1.zip › Figure 1-Source Data 1/wb1 090721 4min 2021.07.09 CD9 CD63labelled.tif]

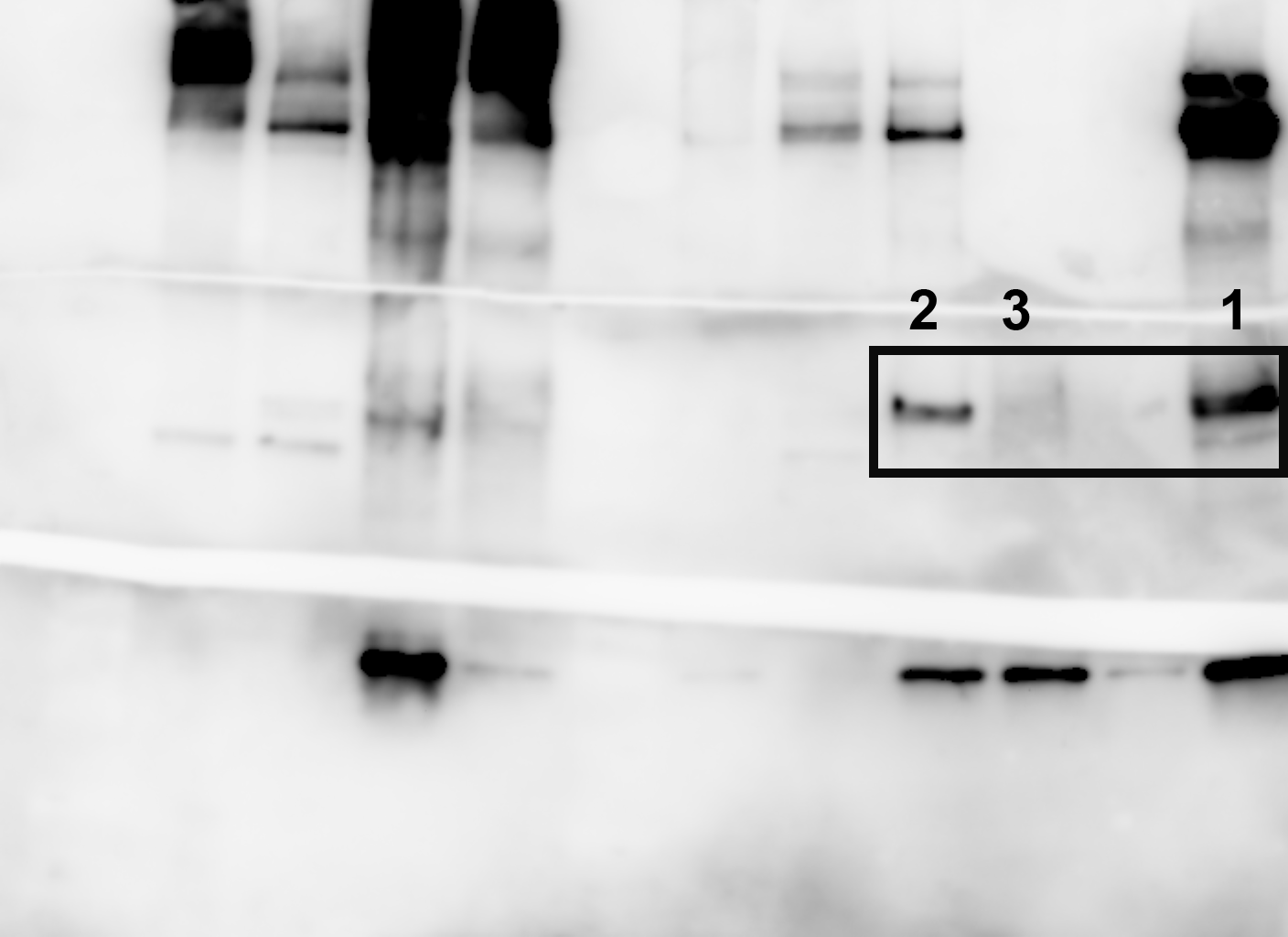

Supplement: Figure 1—source data 1. [file elife-99172-fig1-data1.zip › Figure 1-Source Data 1/wb4 160721 7min 2021.07.16_TUBlabelled.tif]

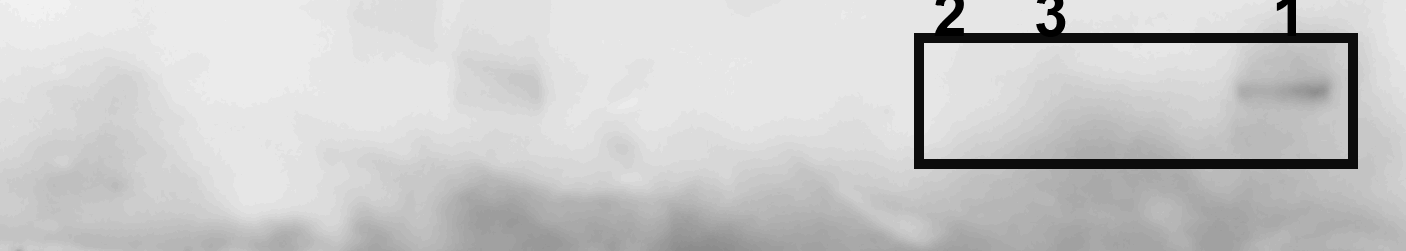

Supplement: Figure 1—source data 1. [file elife-99172-fig1-data1.zip › Figure 1-Source Data 1/wb1 090721 4min 2021.07.09_GM130labelled.tif]

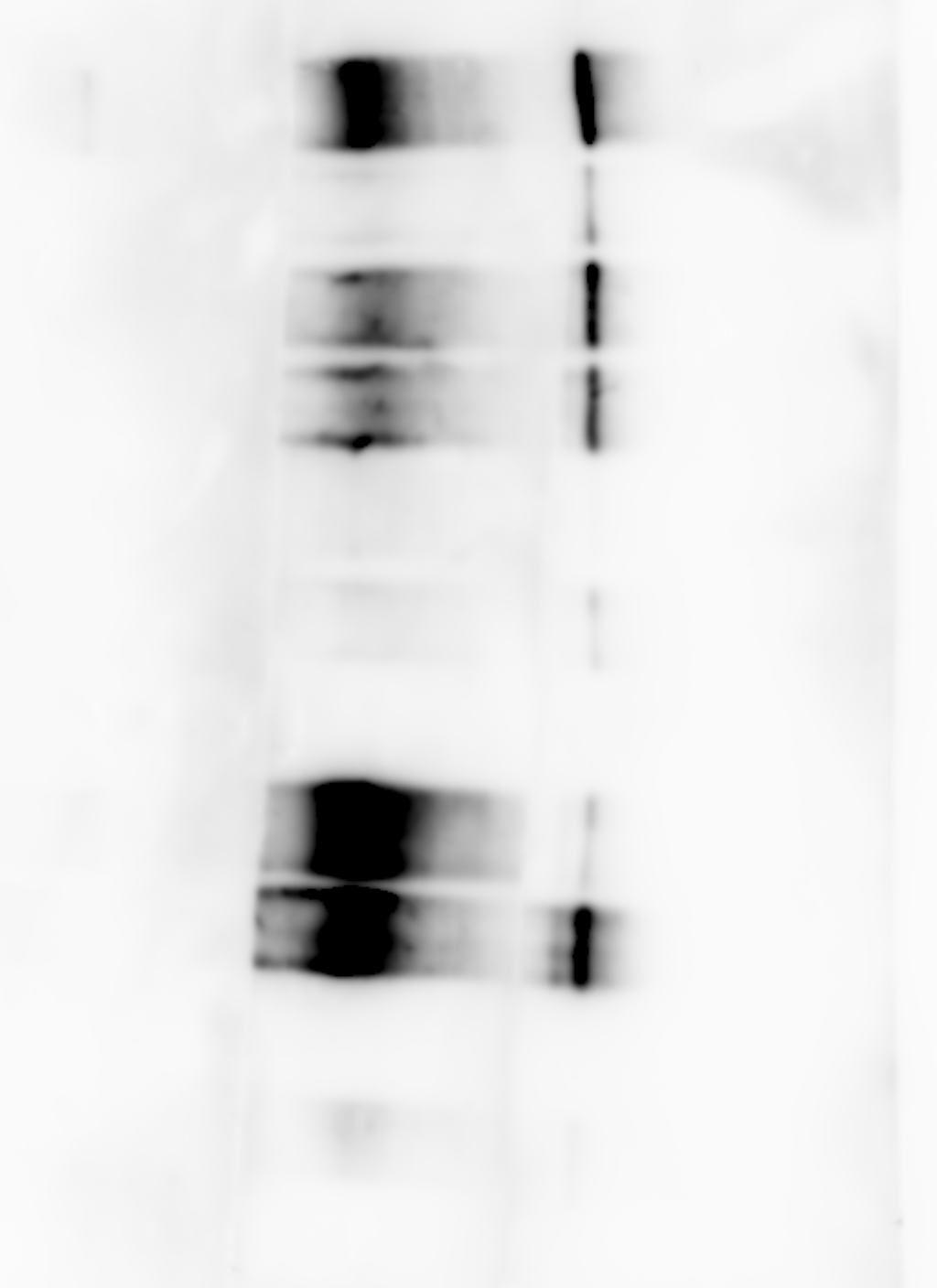

Supplement: Figure 1—source data 2. [file elife-99172-fig1-data2.zip › Figure 1-Source Data 2/wb1 090721 4min 2021.07.09 CD9 CD63.tif]

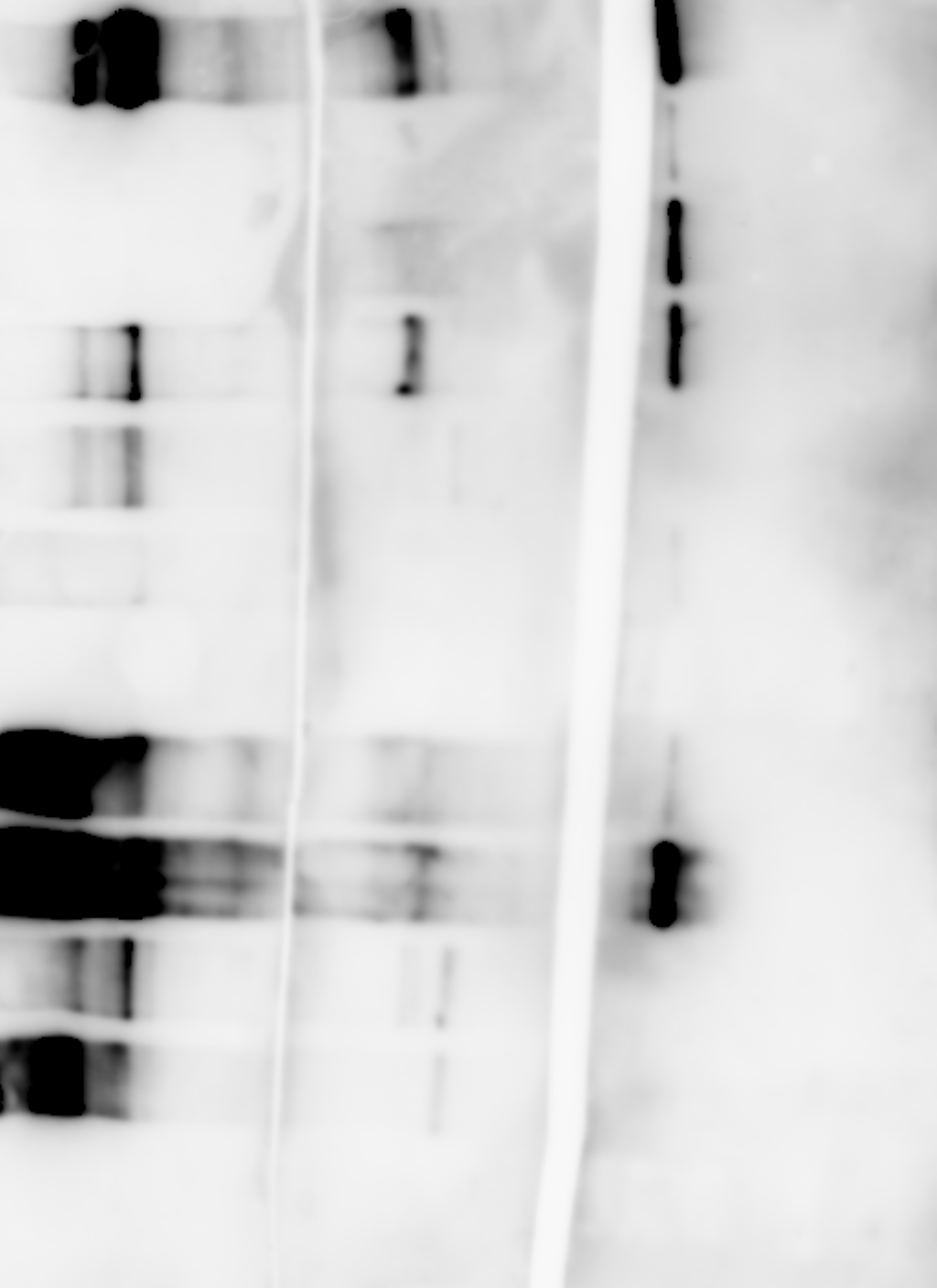

Supplement: Figure 1—source data 2. [file elife-99172-fig1-data2.zip › Figure 1-Source Data 2/wb4 160721 7min 2021.07.16_TUB.tif]

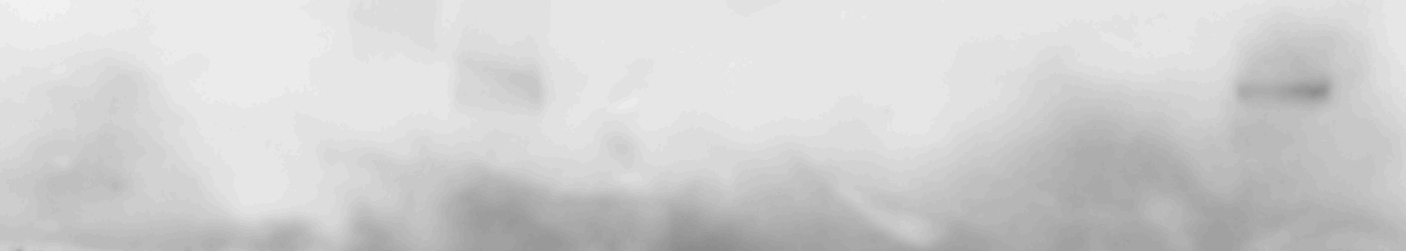

Supplement: Figure 1—source data 2. [file elife-99172-fig1-data2.zip › Figure 1-Source Data 2/wb1 090721 4min 2021.07.09_GM130.tif]

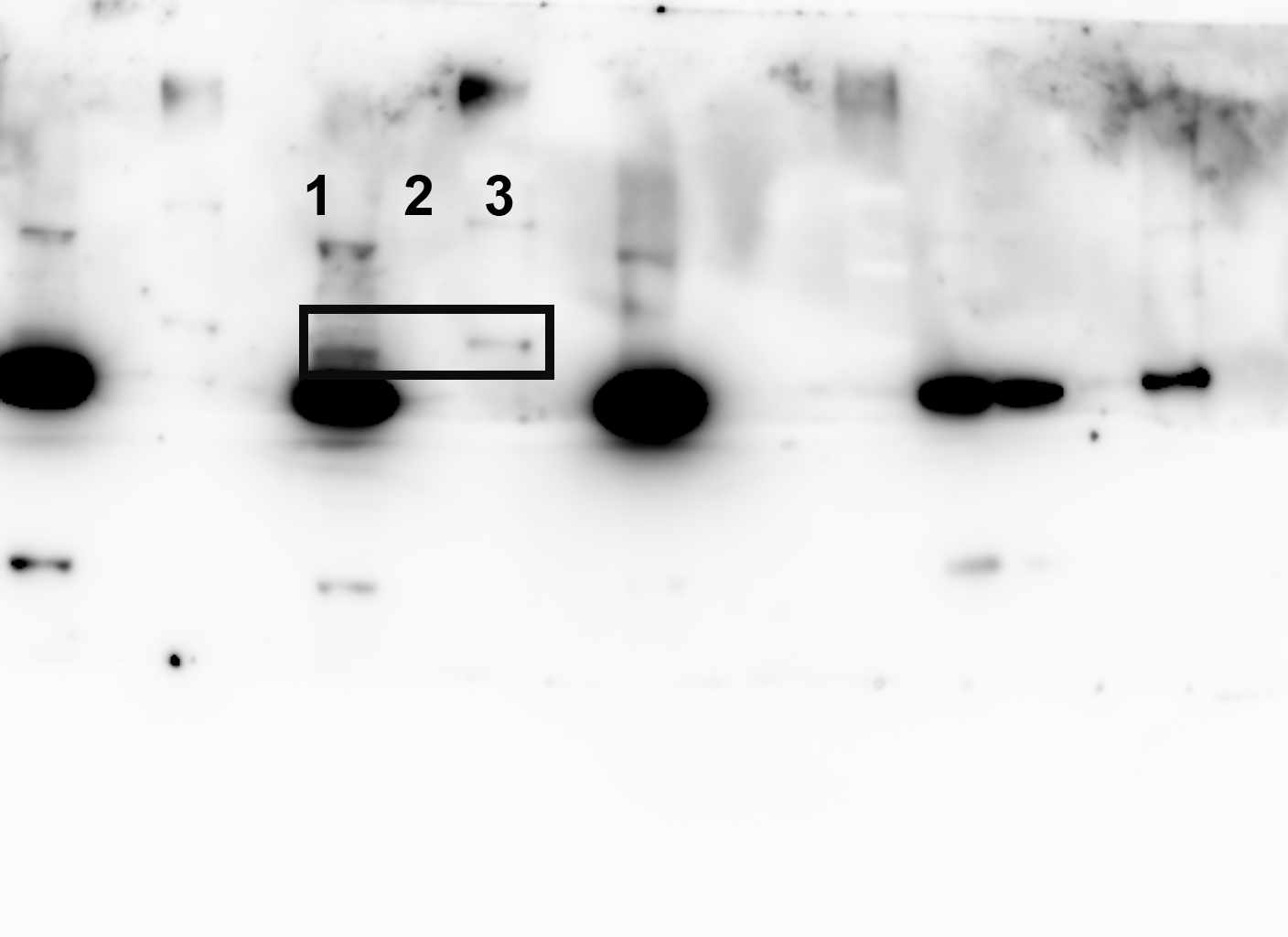

Supplement: Figure 1—figure supplement 1—source data 1. [file elife-99172-fig1-figsupp1-data1.zip › Figure 1-Figure supplement 1F-Source Data 1/wb2 15 min 2023.06.14_alixlabelled.tif]

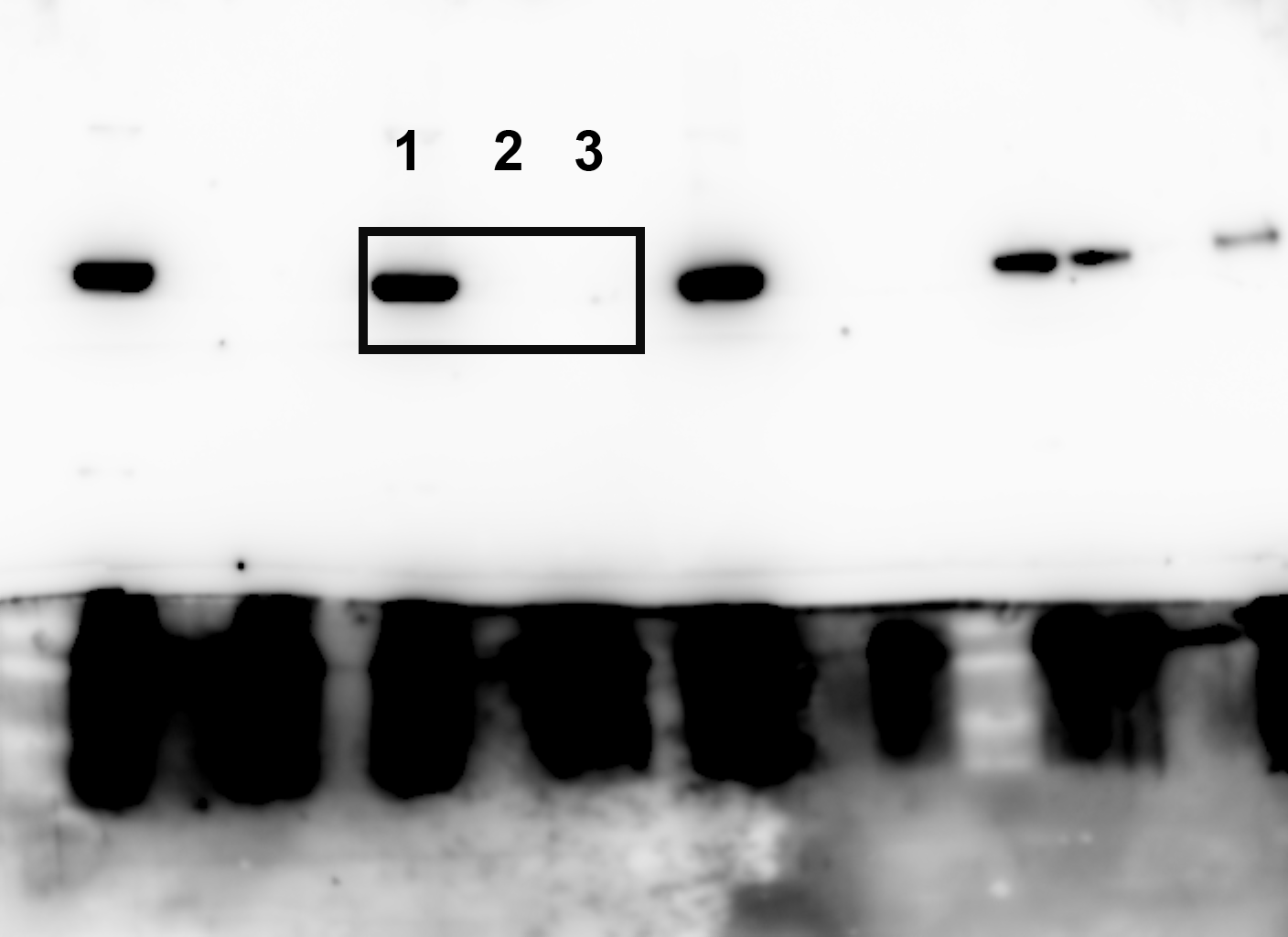

Supplement: Figure 1—figure supplement 1—source data 1. [file elife-99172-fig1-figsupp1-data1.zip › Figure 1-Figure supplement 1F-Source Data 1/wb1 1min 2023.06.13_calnexin labelled.tif]

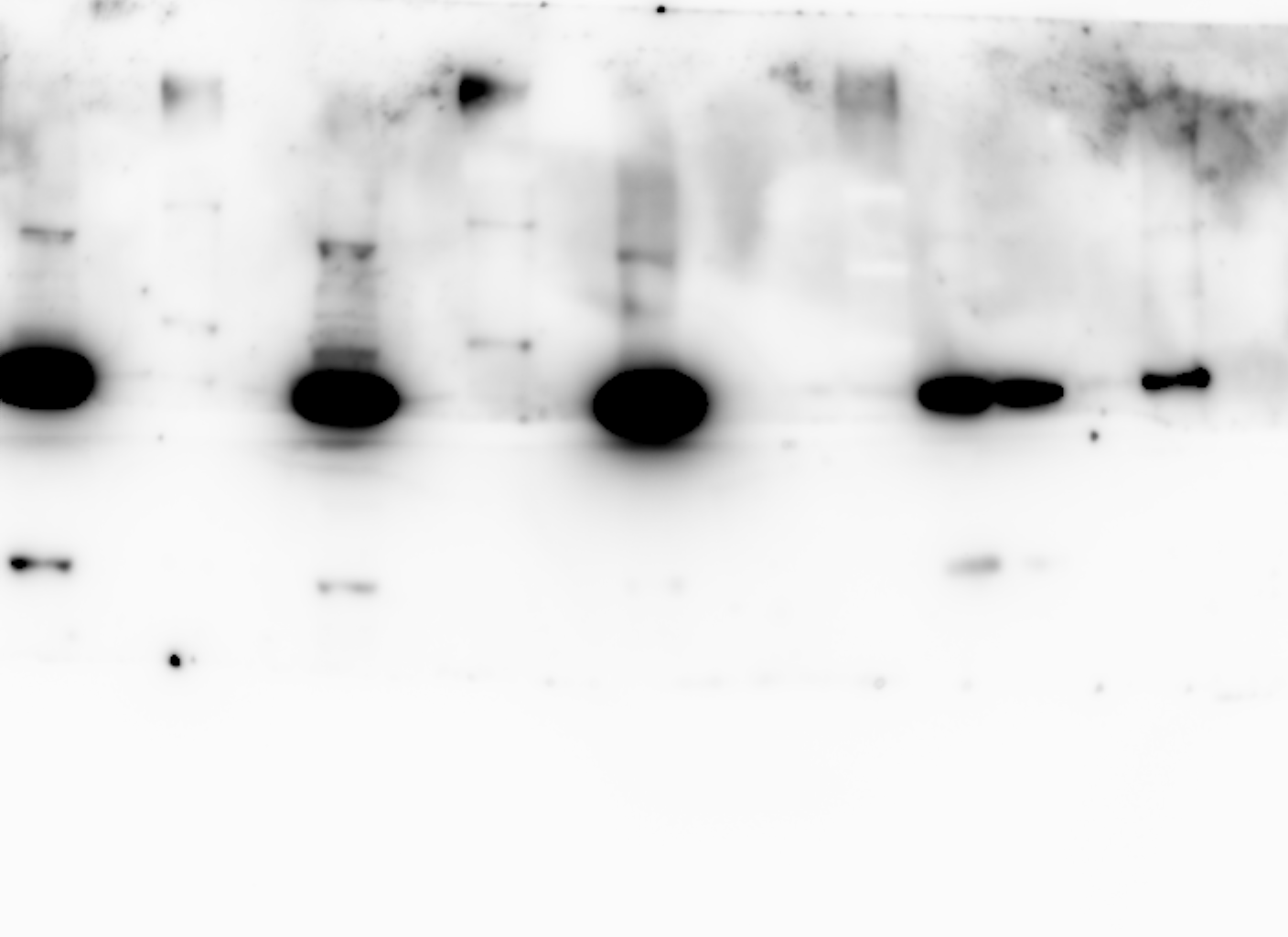

Supplement: Figure 1—figure supplement 1—source data 2. [file elife-99172-fig1-figsupp1-data2.zip › Figure 1-figure supplement 1F-Source Data 2/wb2 15 min 2023.06.14_alix.tif]

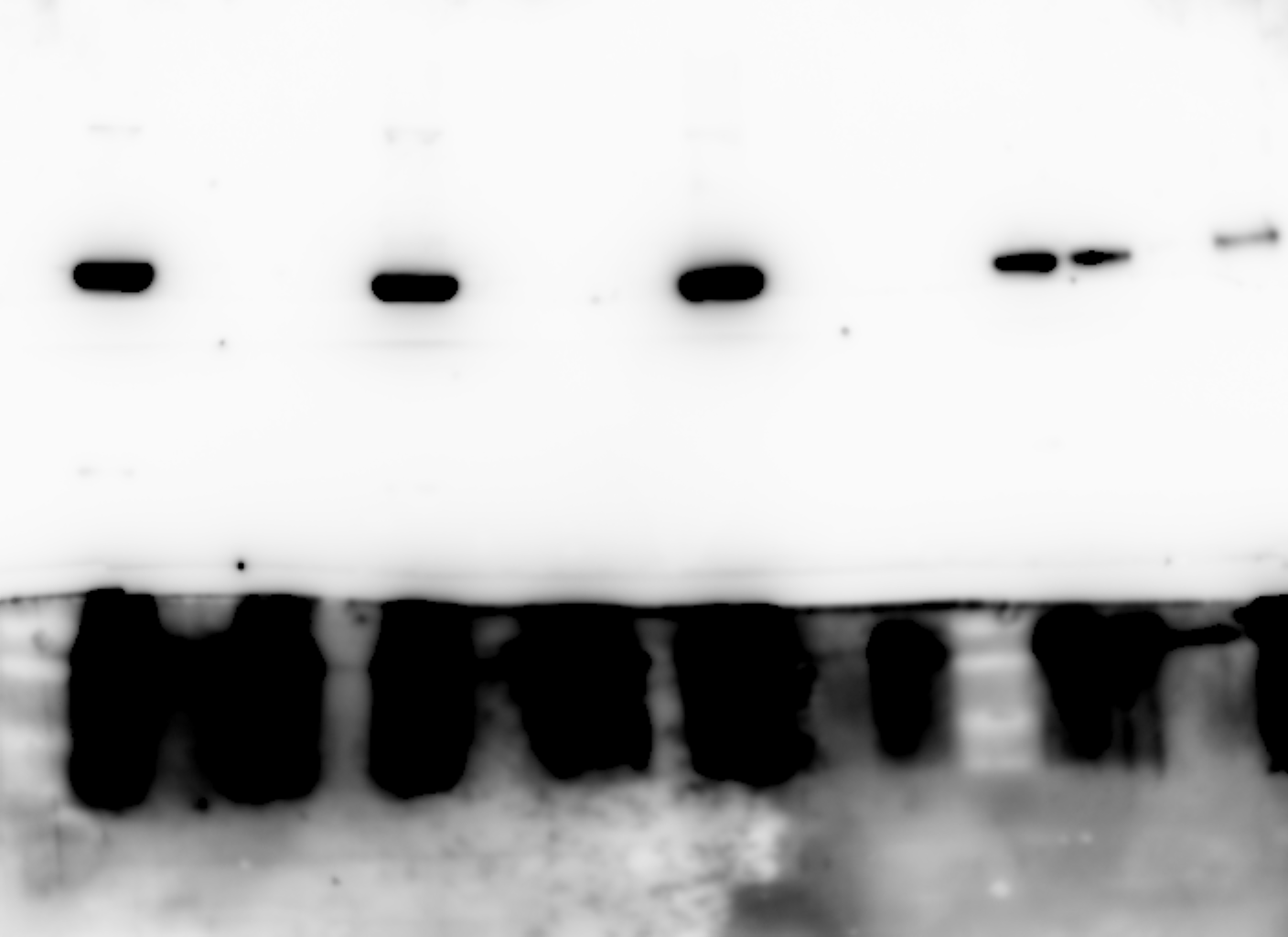

Supplement: Figure 1—figure supplement 1—source data 2. [file elife-99172-fig1-figsupp1-data2.zip › Figure 1-figure supplement 1F-Source Data 2/wb1 1min 2023.06.13_calnexin.tif]

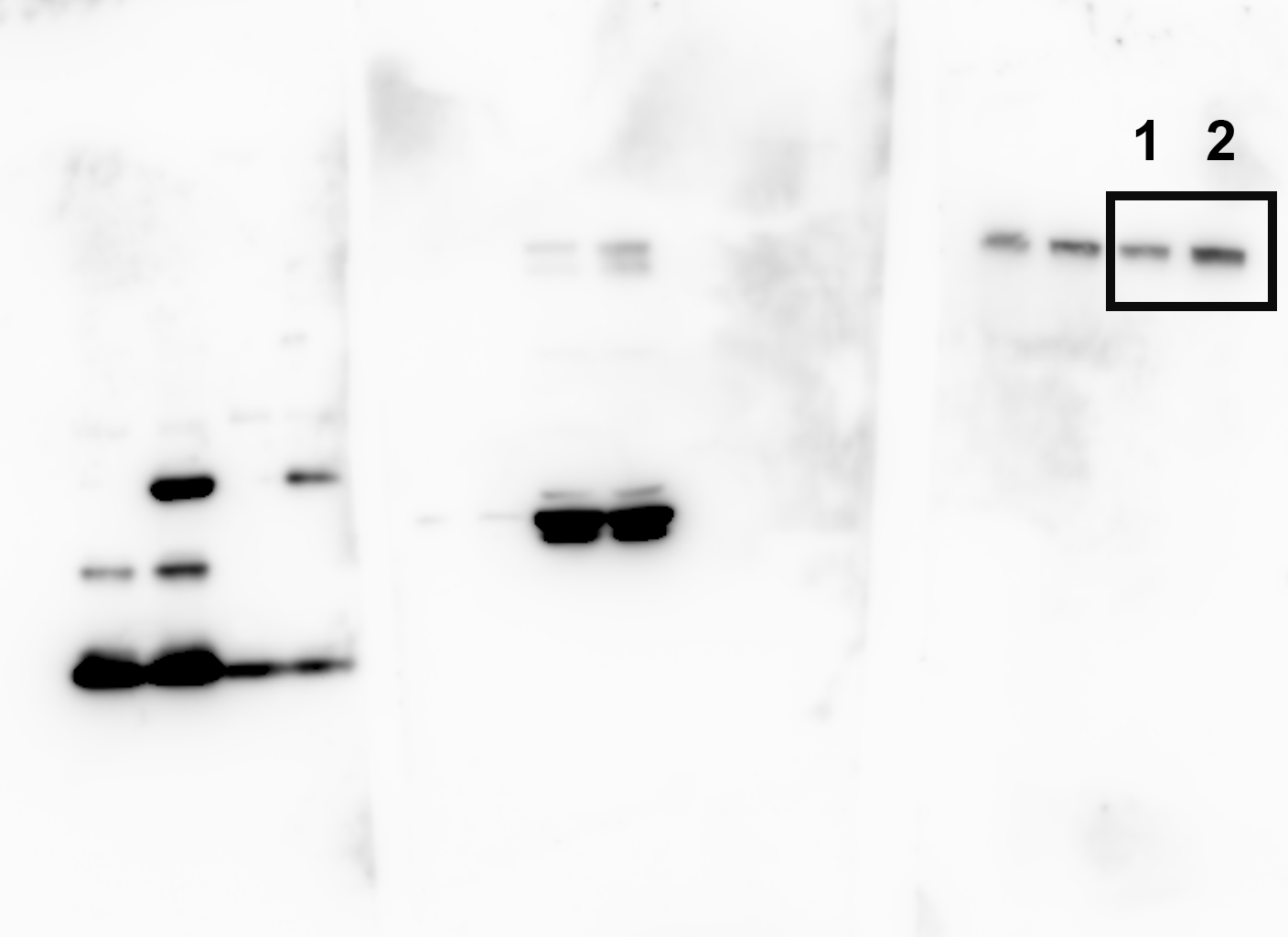

Supplement: Figure 2—figure supplement 1—source data 1. [file elife-99172-fig2-figsupp1-data1.zip › Figure 2-figure supplement 1G-Source Data 1/wb3 5min 2022.06.23_EGFRlabelled.tif]

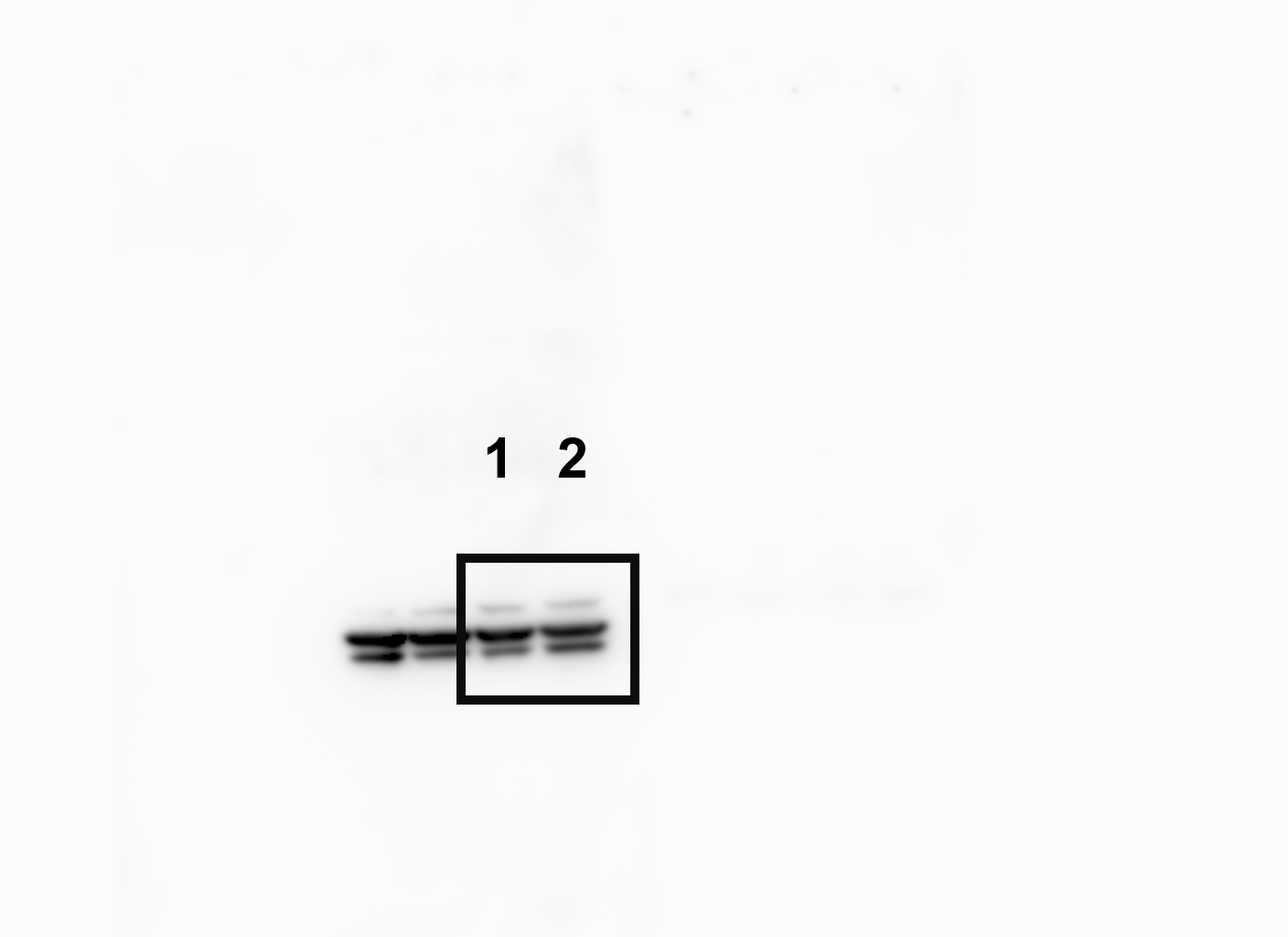

Supplement: Figure 2—figure supplement 1—source data 1. [file elife-99172-fig2-figsupp1-data1.zip › Figure 2-figure supplement 1G-Source Data 1/wb3.1 1min 2022.06.23_ANXA2labelled..tif]

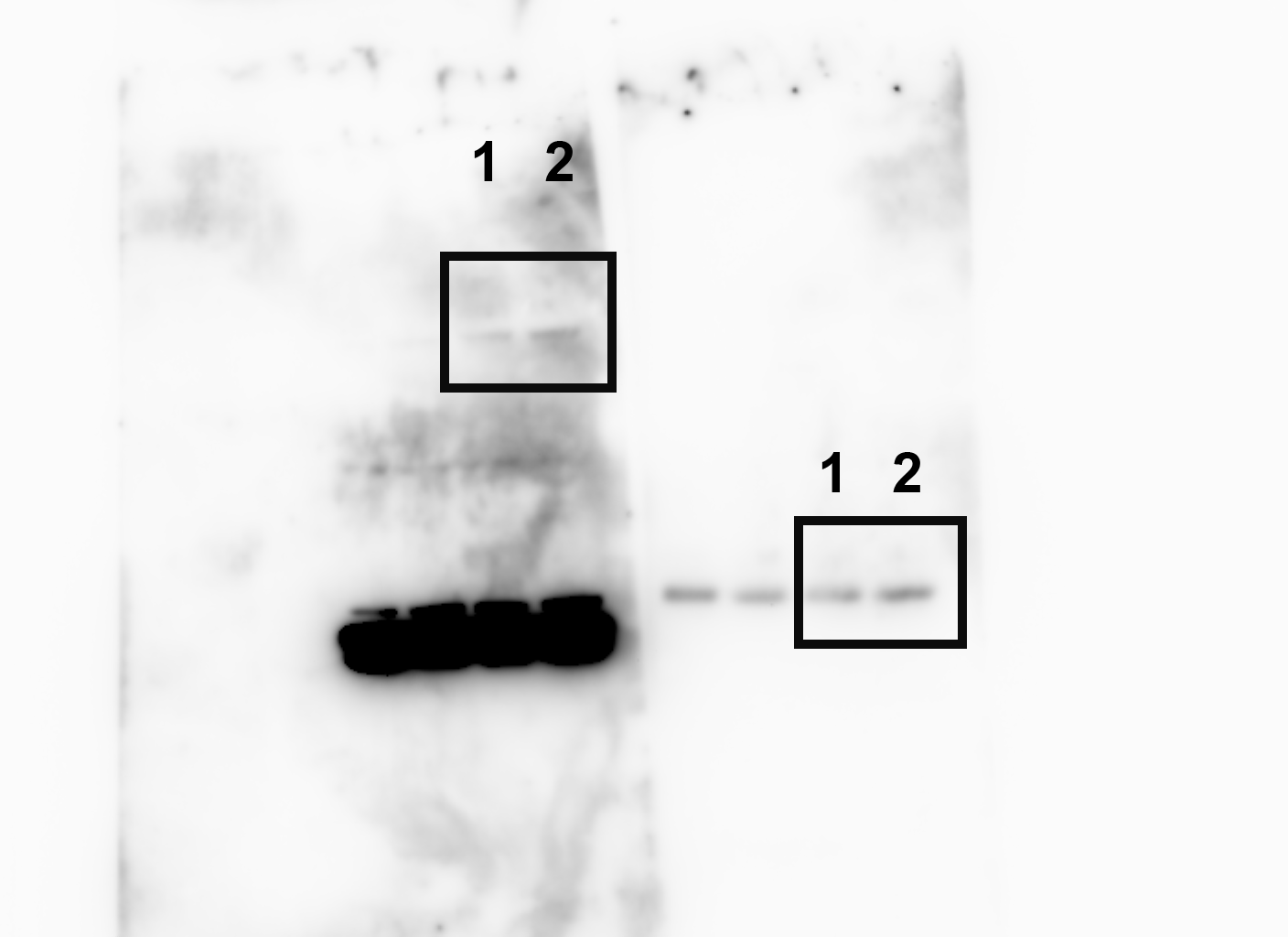

Supplement: Figure 2—figure supplement 1—source data 1. [file elife-99172-fig2-figsupp1-data1.zip › Figure 2-figure supplement 1G-Source Data 1/wb3.1 10min 2022.06.23_INTb4_Cx43labelled.tif]

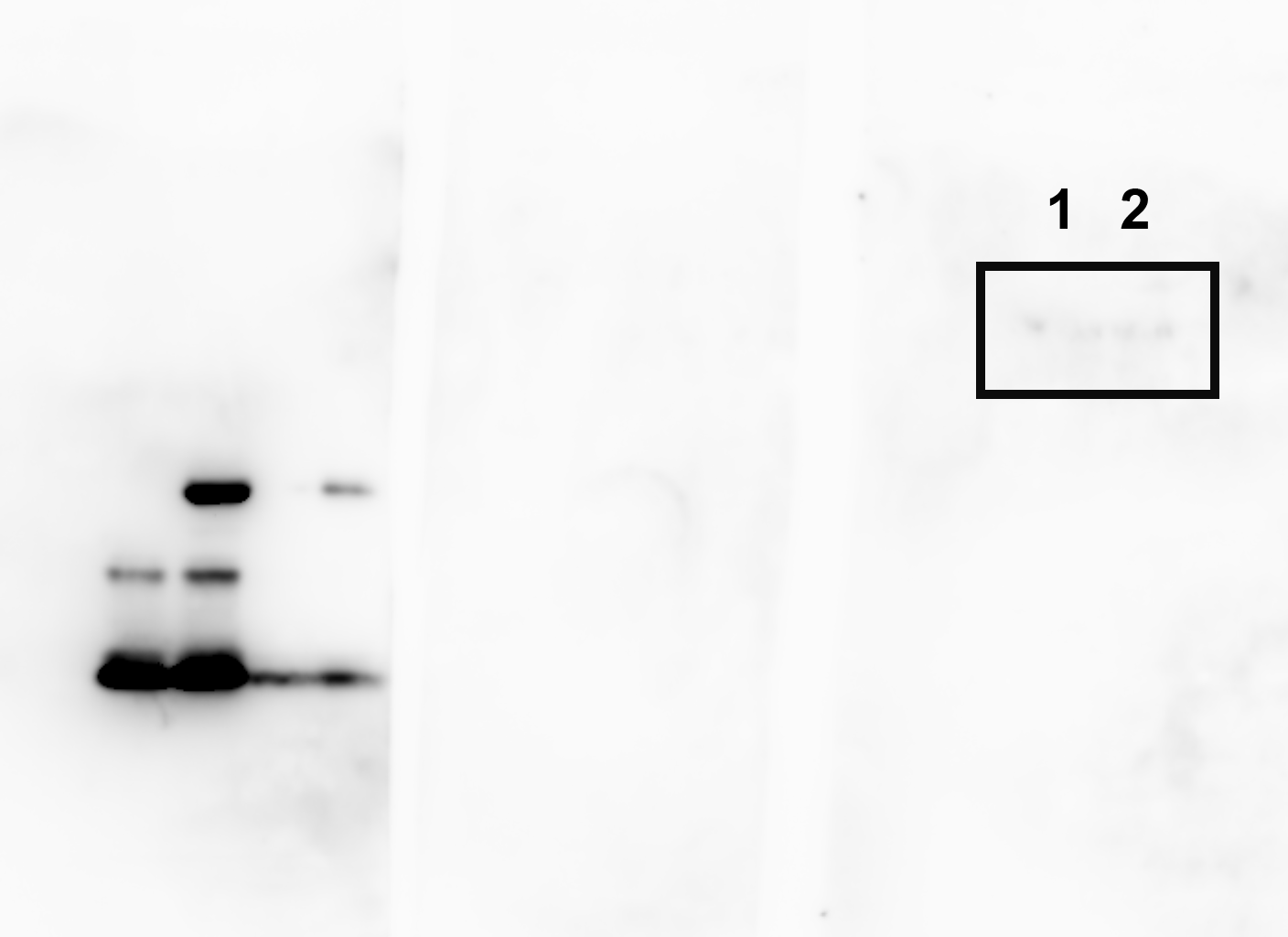

Supplement: Figure 2—figure supplement 1—source data 1. [file elife-99172-fig2-figsupp1-data1.zip › Figure 2-figure supplement 1G-Source Data 1/wb 1 -dtt 5 min 2022.06.21_INTa4labelled.tif]

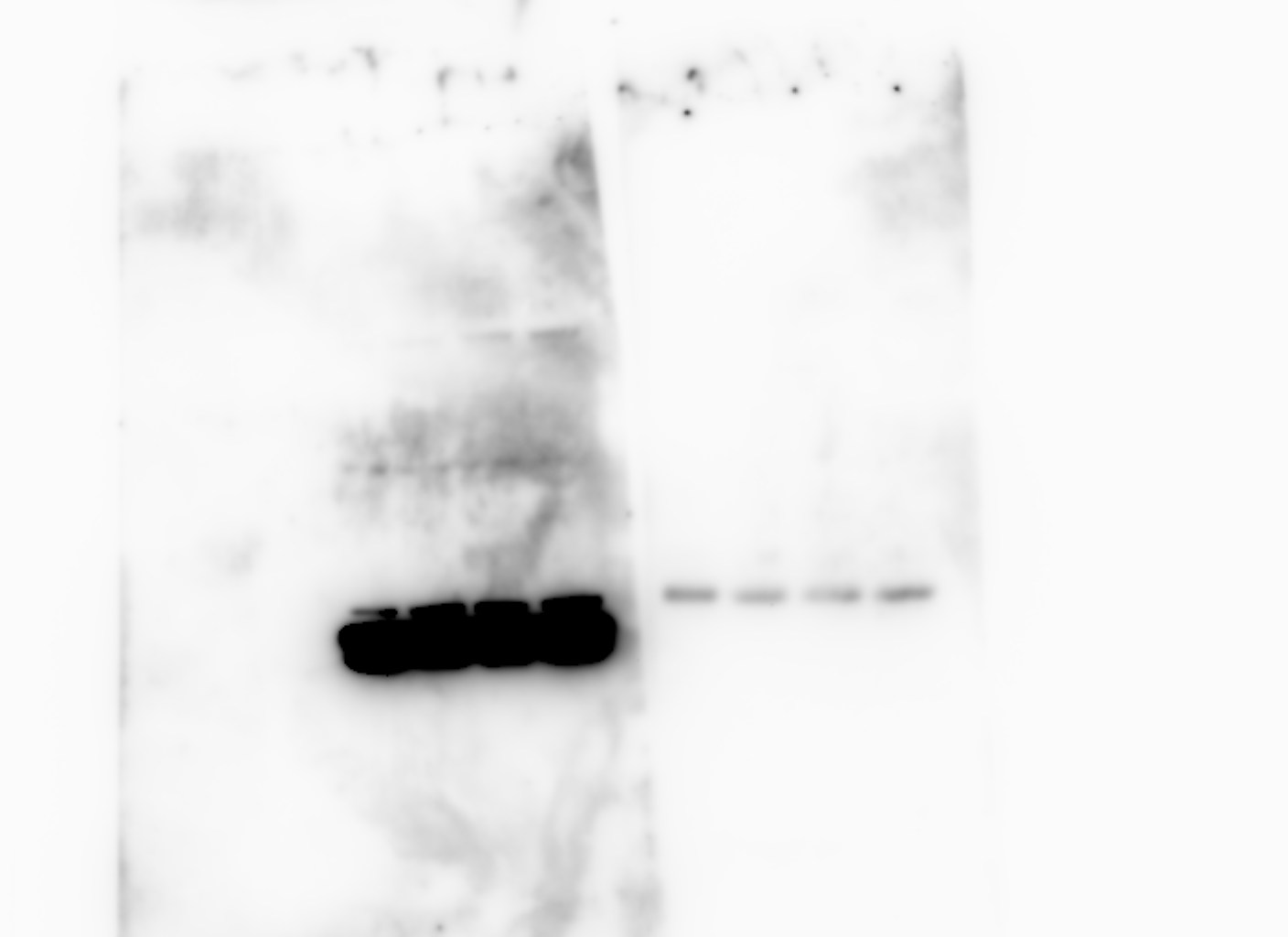

Supplement: Figure 2—figure supplement 1—source data 2. [file elife-99172-fig2-figsupp1-data2.zip › Figure 2-figure supplement 1G-Source Data 2/wb3.1 10min 2022.06.23_INTb4_Cx43.tif]

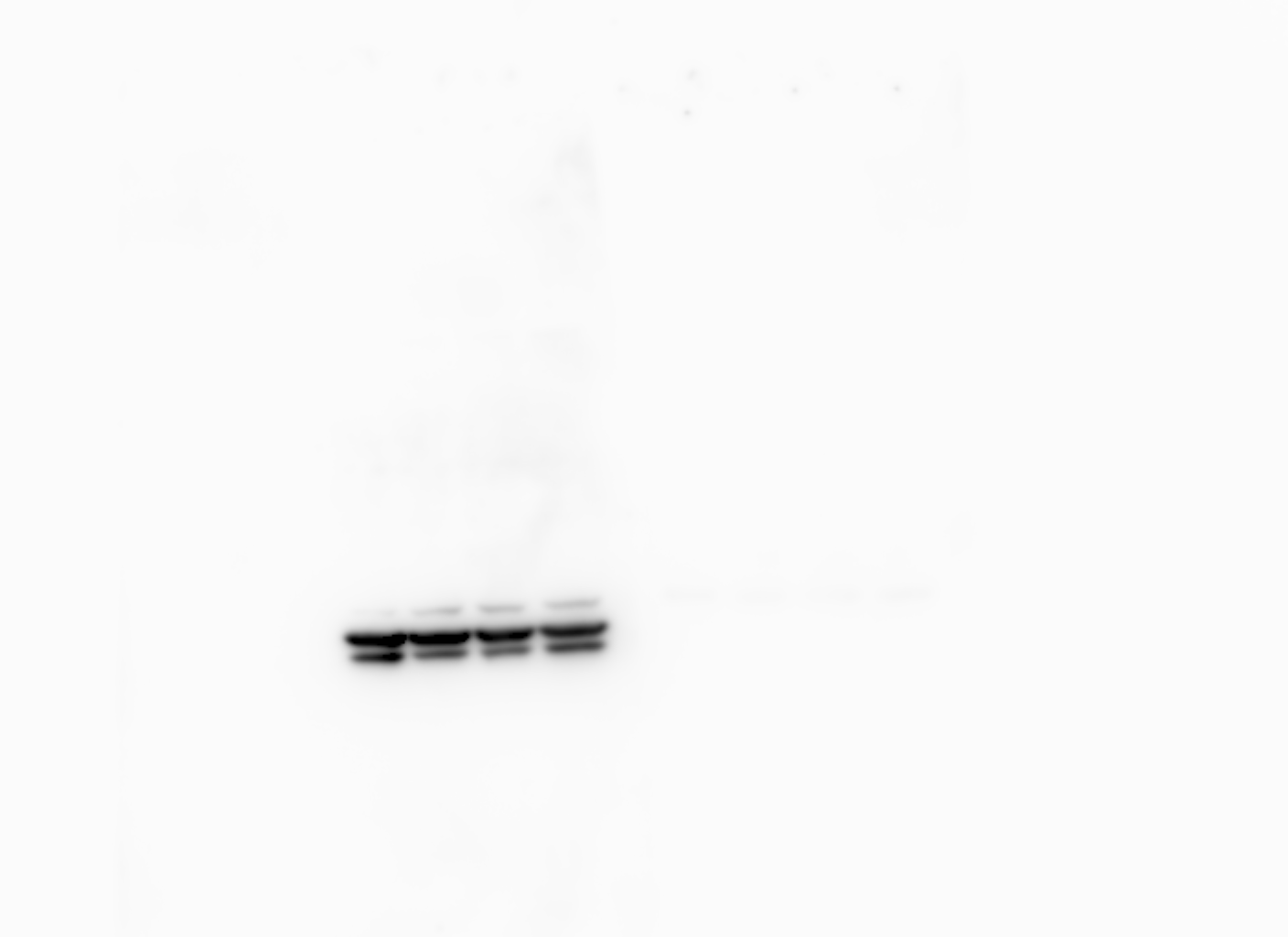

Supplement: Figure 2—figure supplement 1—source data 2. [file elife-99172-fig2-figsupp1-data2.zip › Figure 2-figure supplement 1G-Source Data 2/wb3.1 1min 2022.06.23_ANXA2..tif]

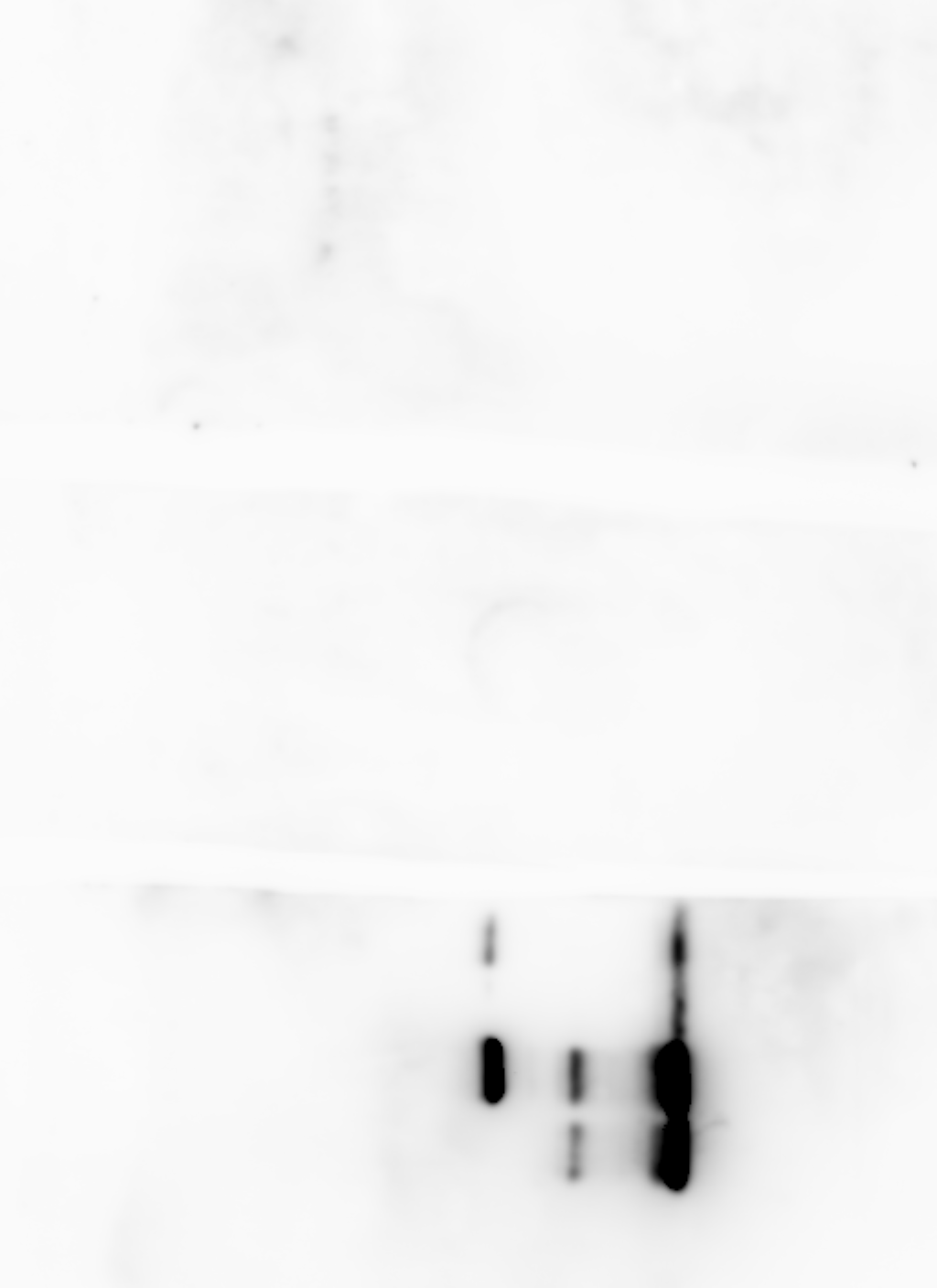

Supplement: Figure 2—figure supplement 1—source data 2. [file elife-99172-fig2-figsupp1-data2.zip › Figure 2-figure supplement 1G-Source Data 2/wb 1 -dtt 5 min 2022.06.21_INTa4.tif]

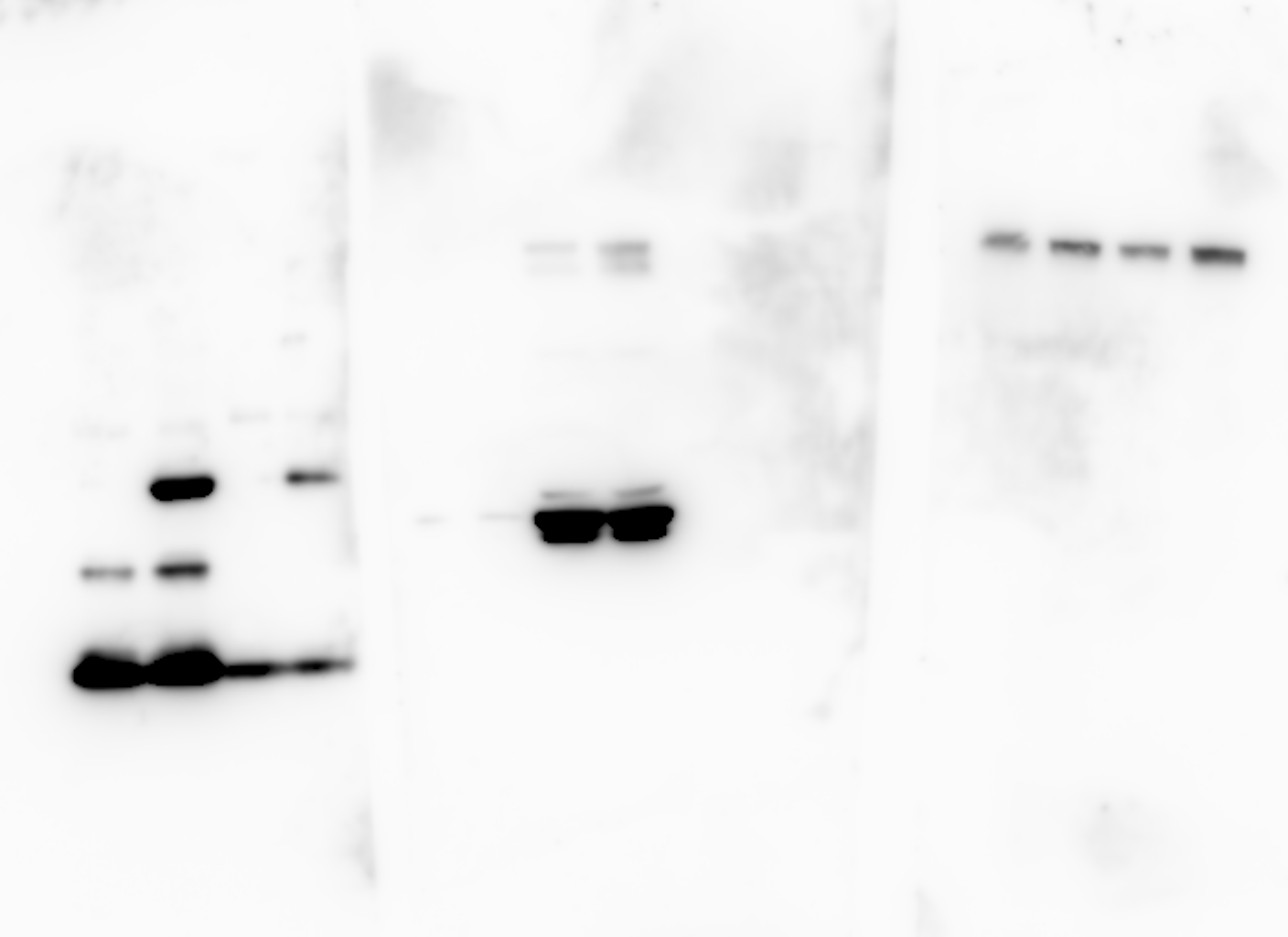

Supplement: Figure 2—figure supplement 1—source data 2. [file elife-99172-fig2-figsupp1-data2.zip › Figure 2-figure supplement 1G-Source Data 2/wb3 5min 2022.06.23_EGFR.tif]

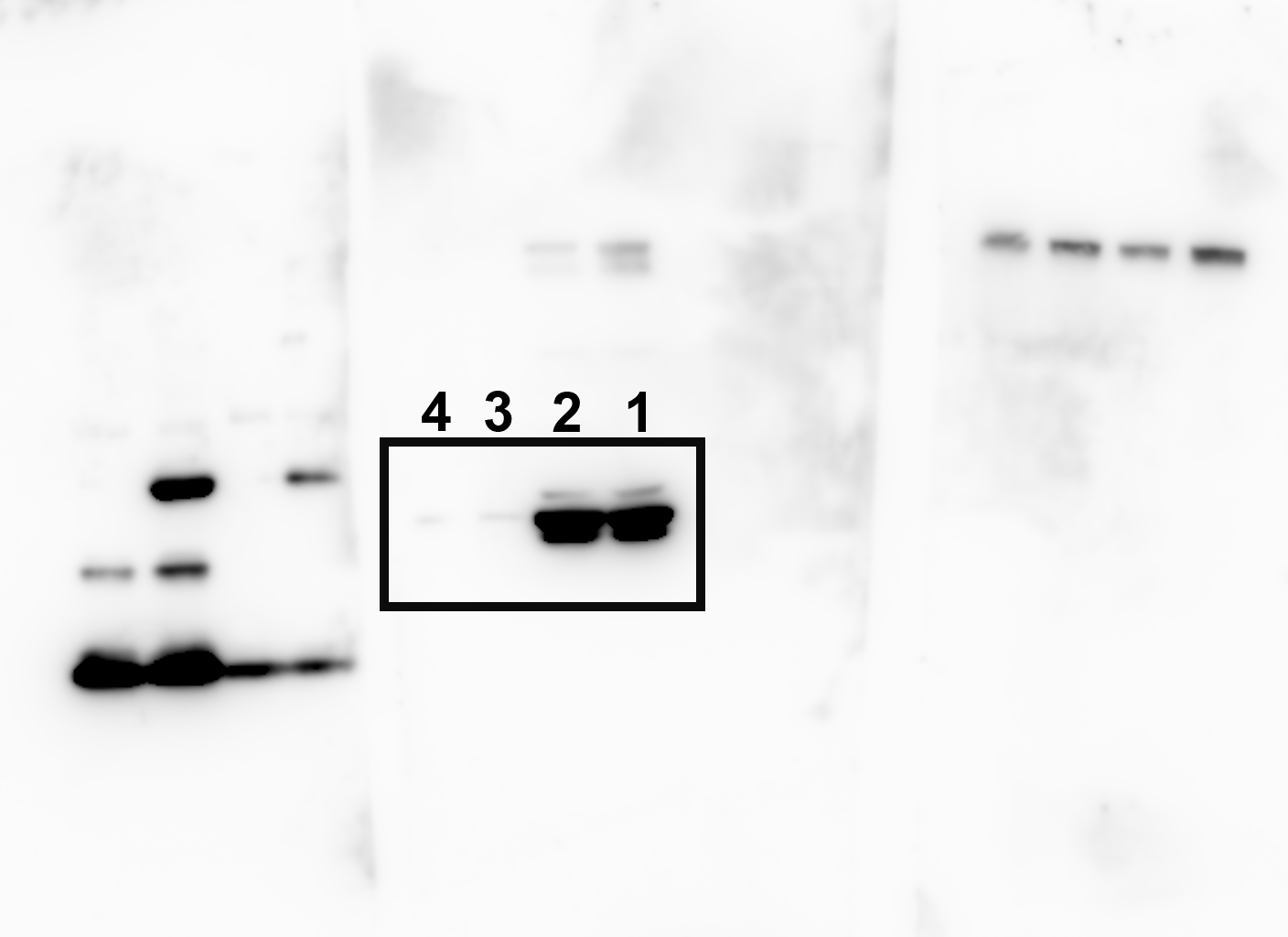

Supplement: Figure 2—figure supplement 1—source data 3. [file elife-99172-fig2-figsupp1-data3.zip › Figure 2-figure supplement 1H-Source Data 1/wb3 5min 2022.06.23_ANXA2labelled.tif]

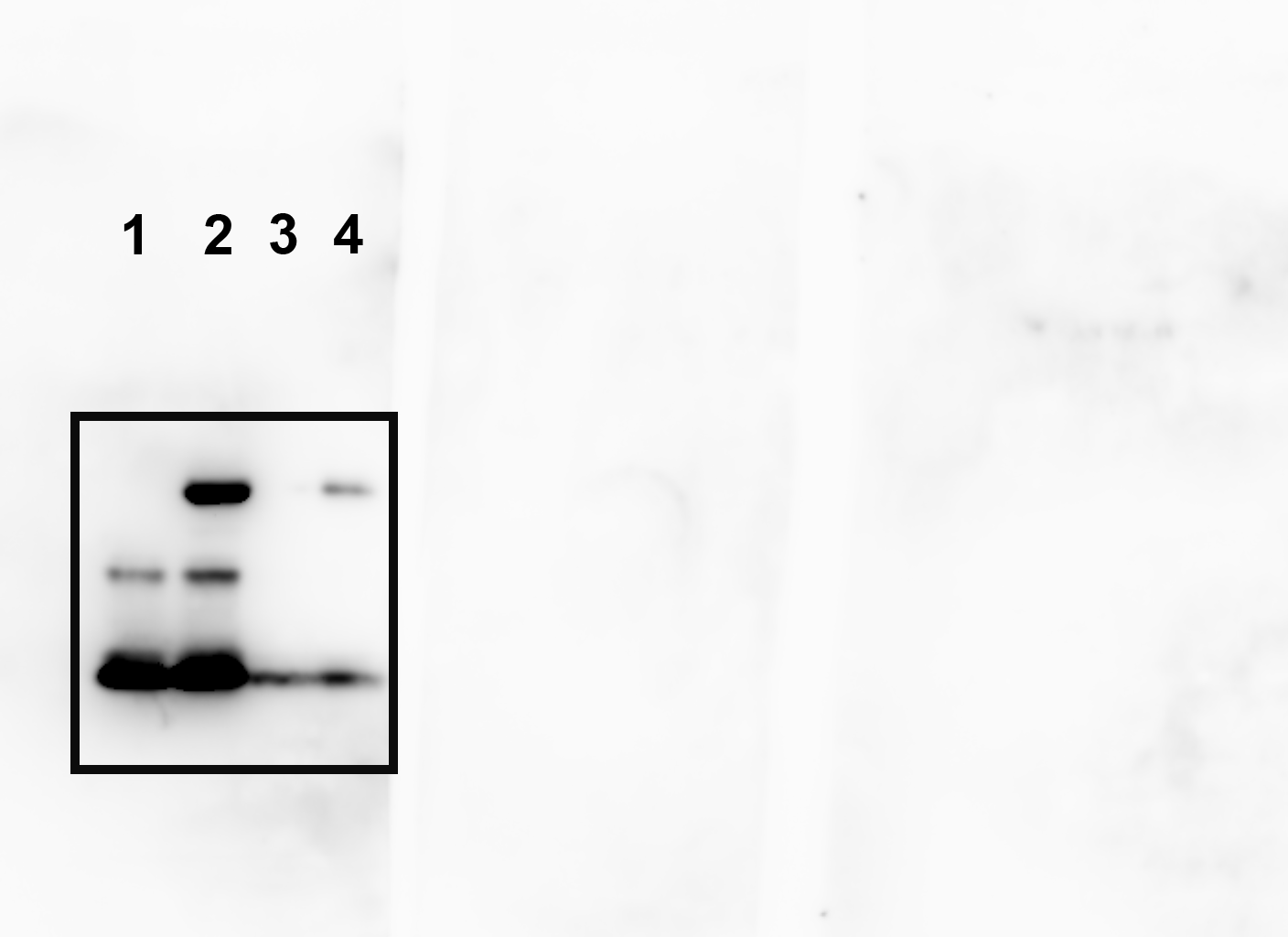

Supplement: Figure 2—figure supplement 1—source data 3. [file elife-99172-fig2-figsupp1-data3.zip › Figure 2-figure supplement 1H-Source Data 1/wb 1 -dtt 5 min 2022.06.21_CD9labelled.tif]

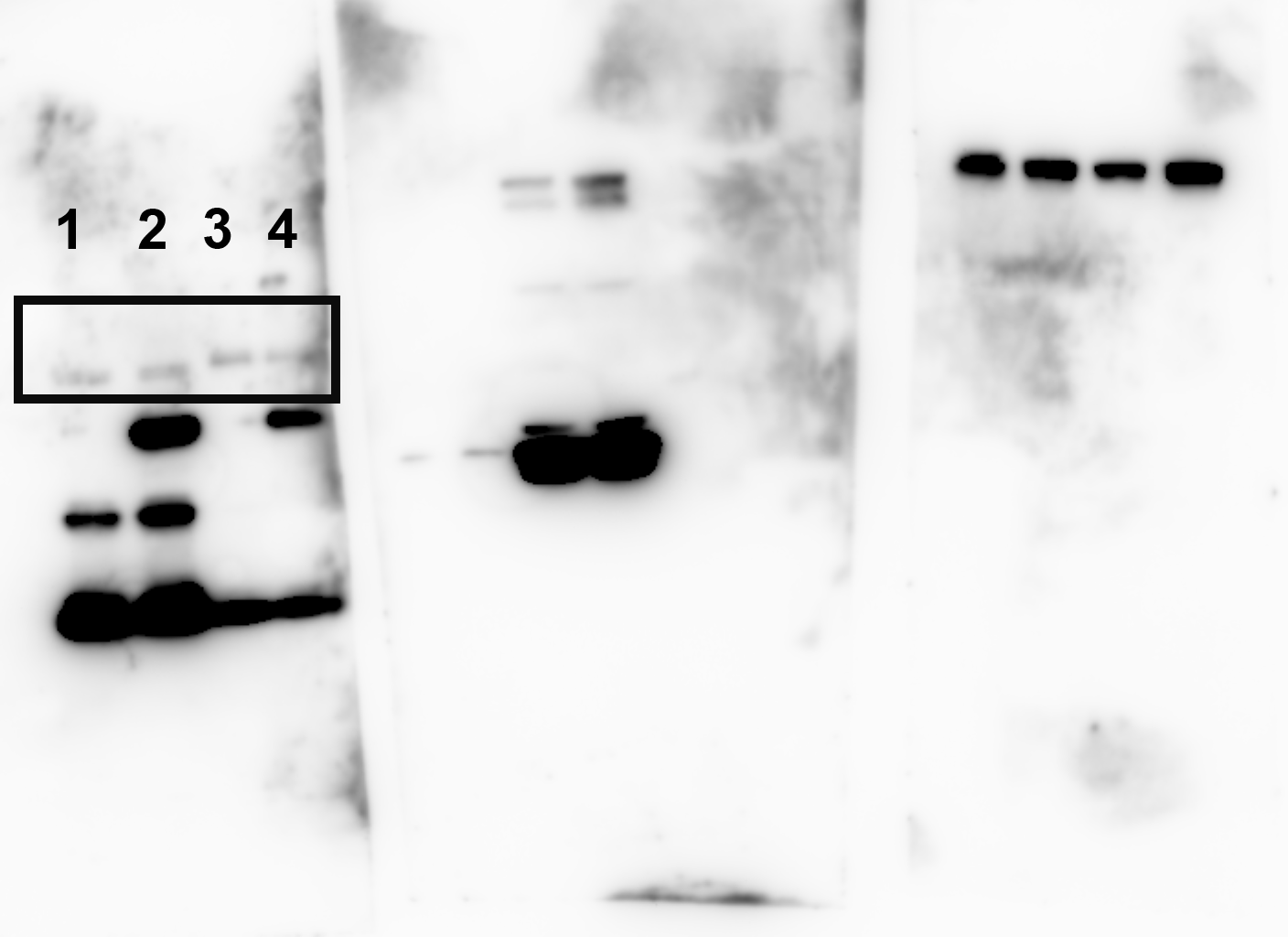

Supplement: Figure 2—figure supplement 1—source data 3. [file elife-99172-fig2-figsupp1-data3.zip › Figure 2-figure supplement 1H-Source Data 1/wb3 15min 2022.06.23_ADAM10labelled.tif]

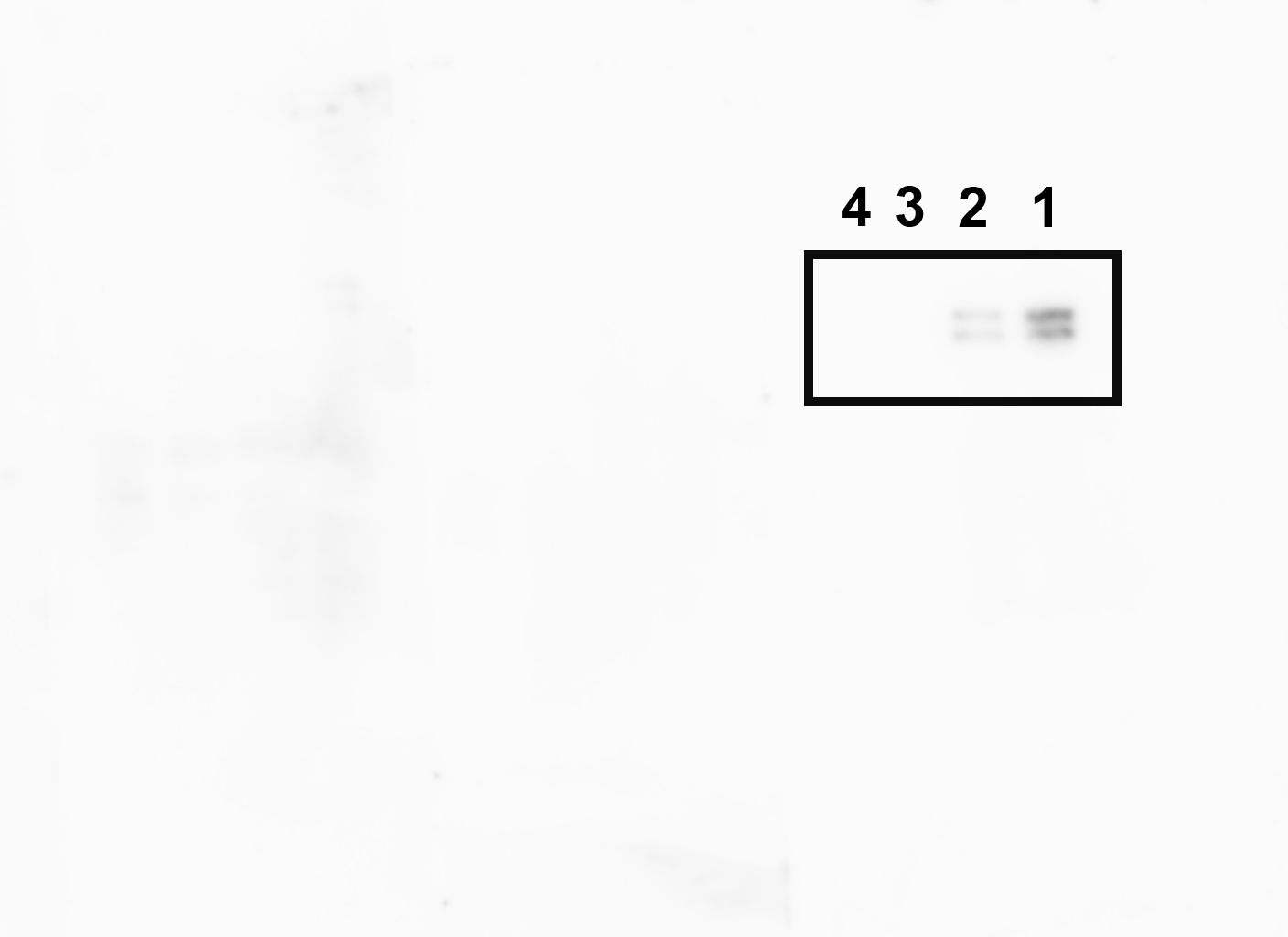

Supplement: Figure 2—figure supplement 1—source data 3. [file elife-99172-fig2-figsupp1-data3.zip › Figure 2-figure supplement 1H-Source Data 1/wb 1 +dtt 7 min 2022.06.21_INTb1labelled.tif]

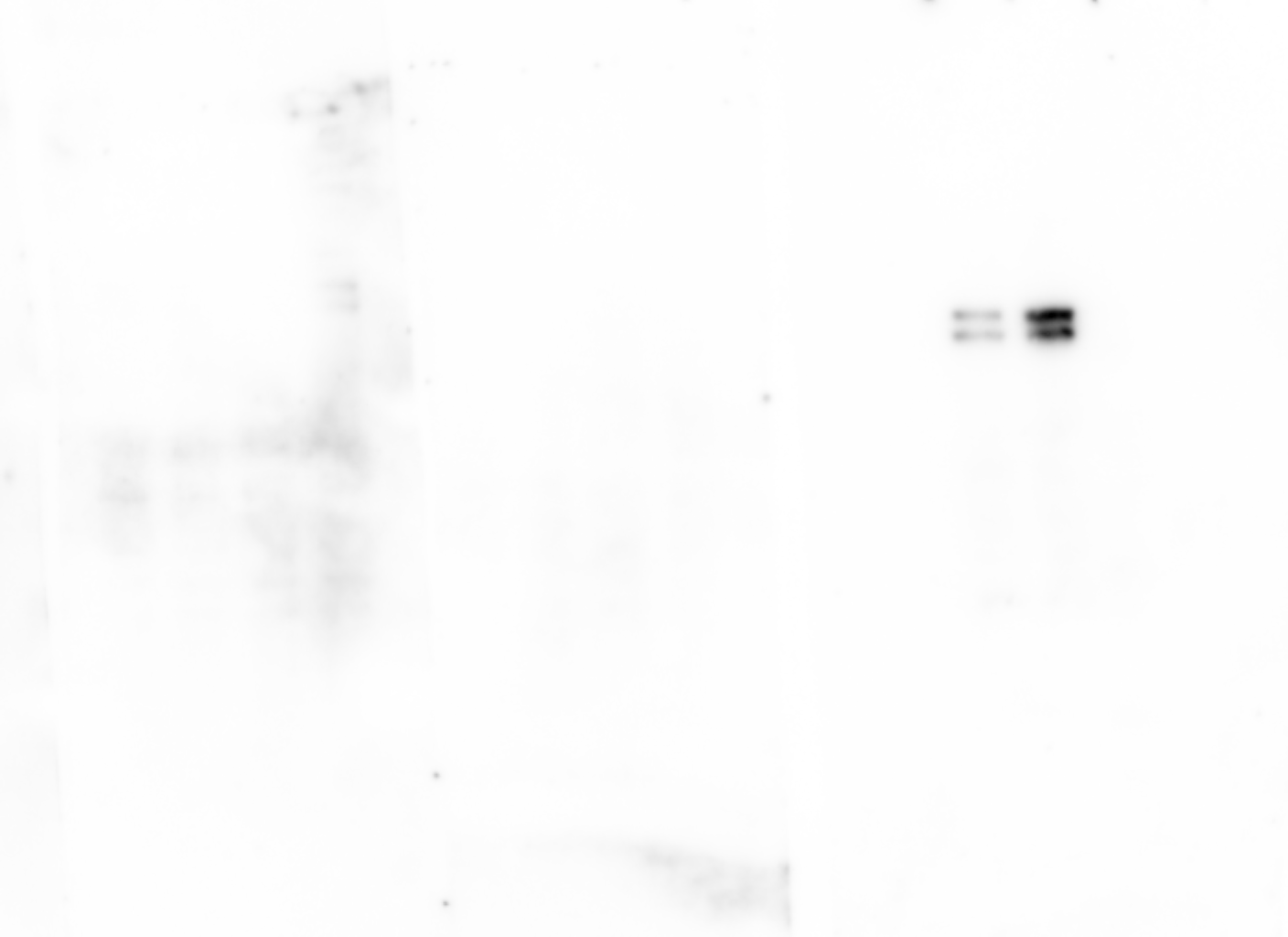

Supplement: Figure 2—figure supplement 1—source data 4. [file elife-99172-fig2-figsupp1-data4.zip › Figure 2-figure supplement 1H-Source Data 2/wb 1 +dtt 7 min 2022.06.21_INTb1.tif]

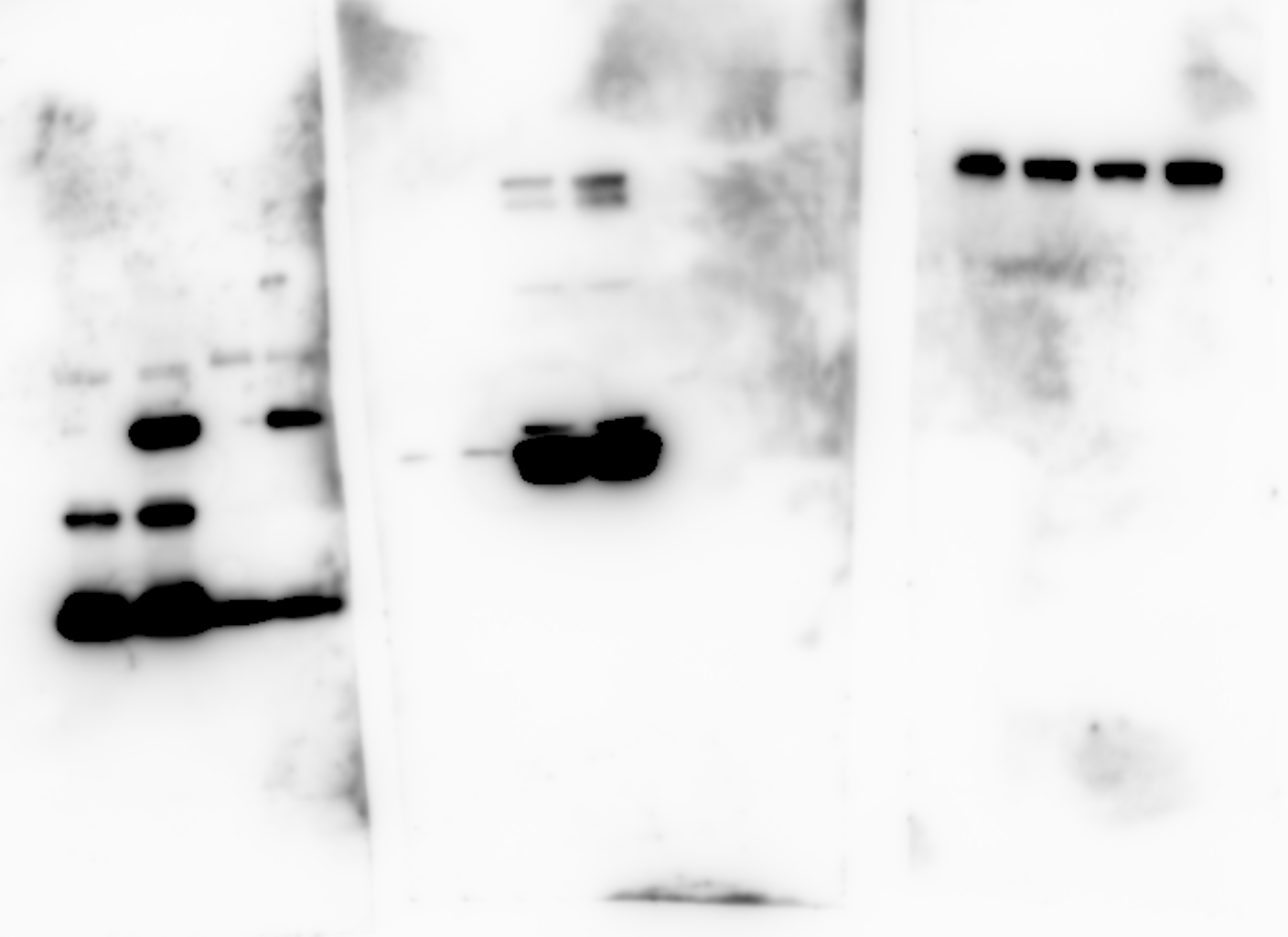

Supplement: Figure 2—figure supplement 1—source data 4. [file elife-99172-fig2-figsupp1-data4.zip › Figure 2-figure supplement 1H-Source Data 2/wb3 15min 2022.06.23_ADAM10.tif]

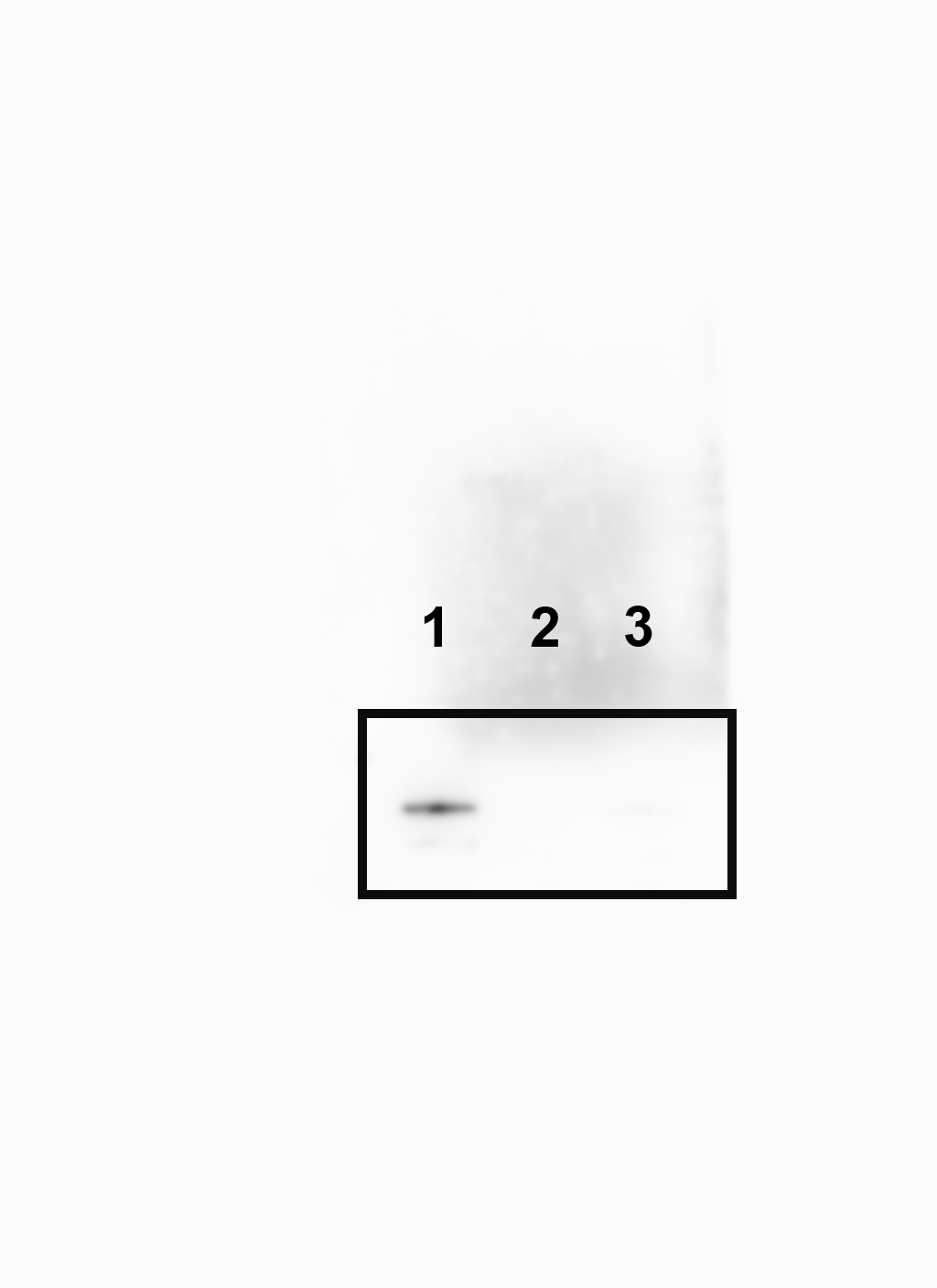

Supplement: Figure 3—figure supplement 1—source data 1. [file elife-99172-fig3-figsupp1-data1.zip › Figure 3-figure supplement 1A-Source Data 1/CD81 of CD9-CD81 KOs AfterExperiments 2021.02.05_12.02.55_Chlabelled.tif]

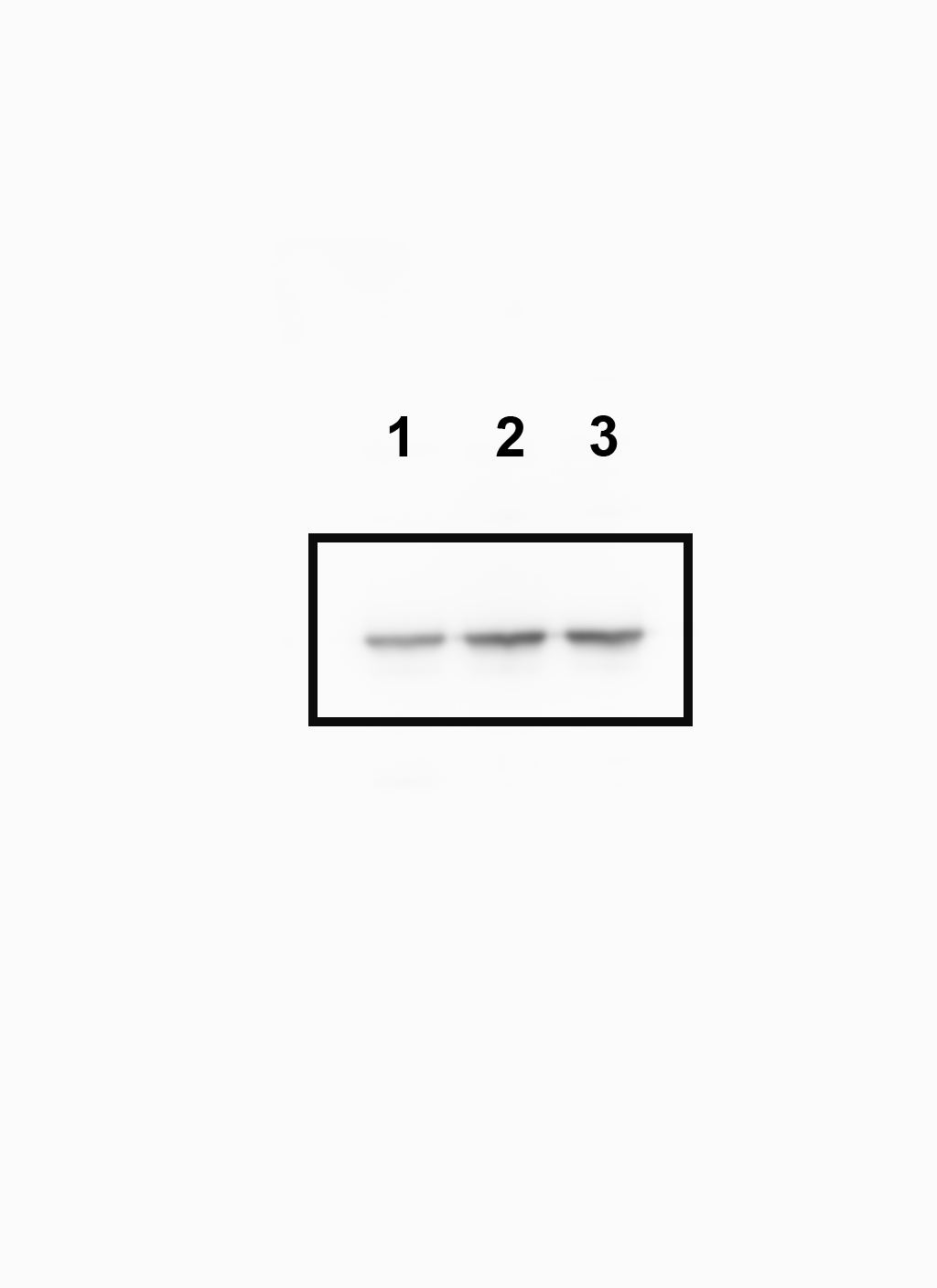

Supplement: Figure 3—figure supplement 1—source data 1. [file elife-99172-fig3-figsupp1-data1.zip › Figure 3-figure supplement 1A-Source Data 1/GAPDH 2 of CD9-CD81 KOs AfterExperiments 2021.02.08_15.44.30_Chlabelled.tif]

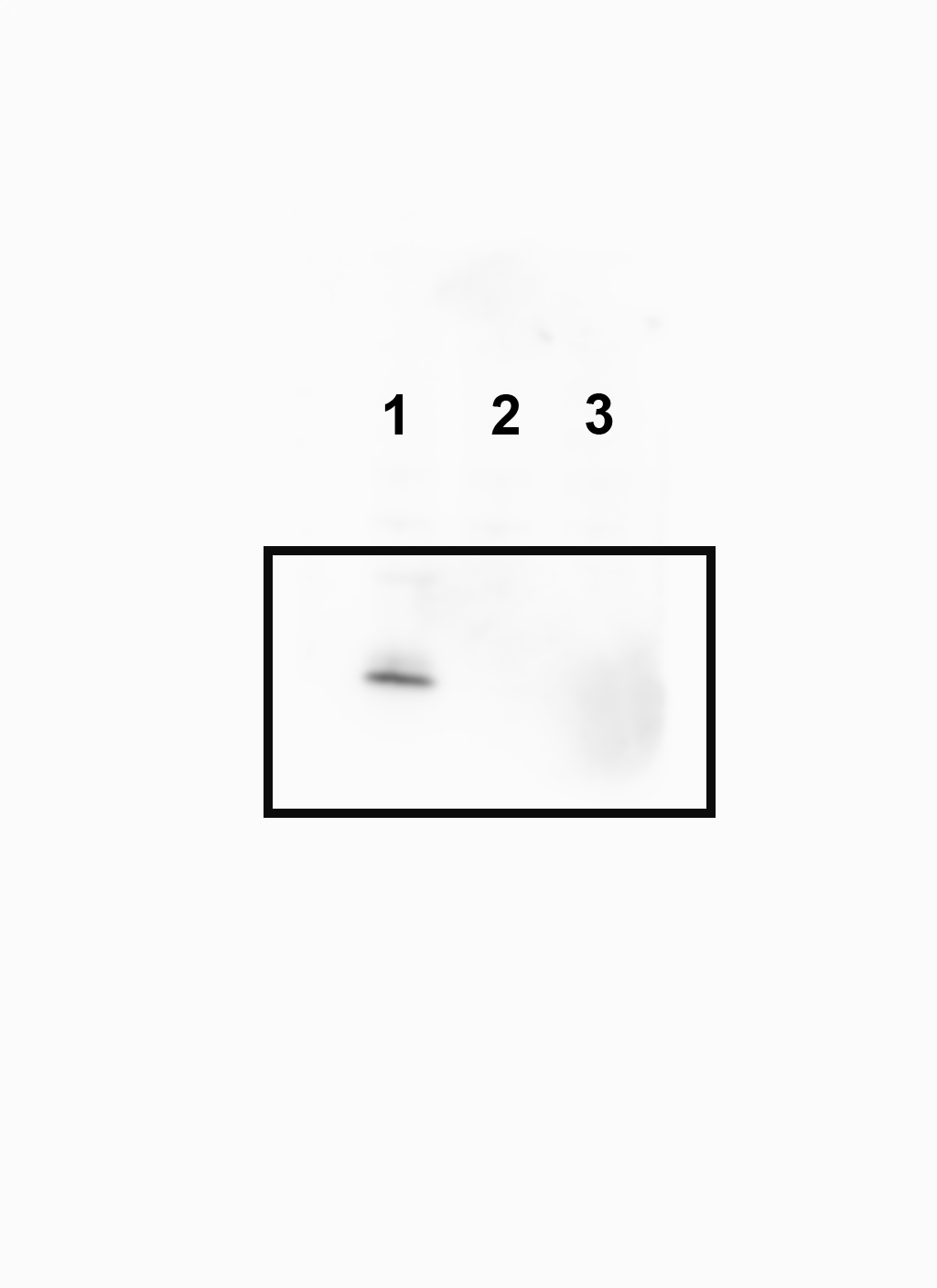

Supplement: Figure 3—figure supplement 1—source data 1. [file elife-99172-fig3-figsupp1-data1.zip › Figure 3-figure supplement 1A-Source Data 1/CD9 of CD9-CD81 KOs AfterExperiments 2021.02.05_1158.51_Chlabelled.tif]

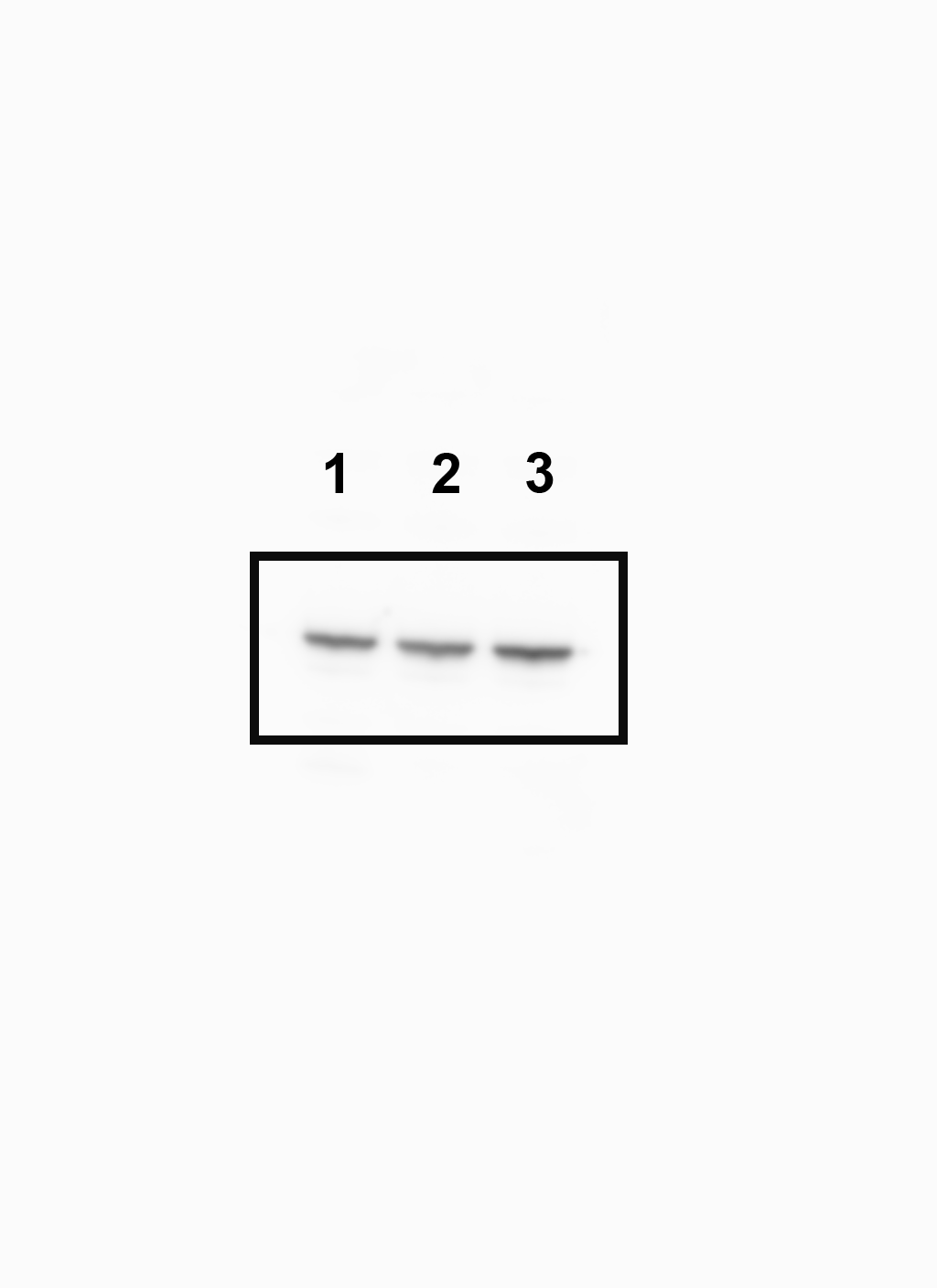

Supplement: Figure 3—figure supplement 1—source data 1. [file elife-99172-fig3-figsupp1-data1.zip › Figure 3-figure supplement 1A-Source Data 1/GAPDH 1 of CD9-CD81 KOs AfterExperiments 2021.02.08_15.41.22_Chlabelled.tif]

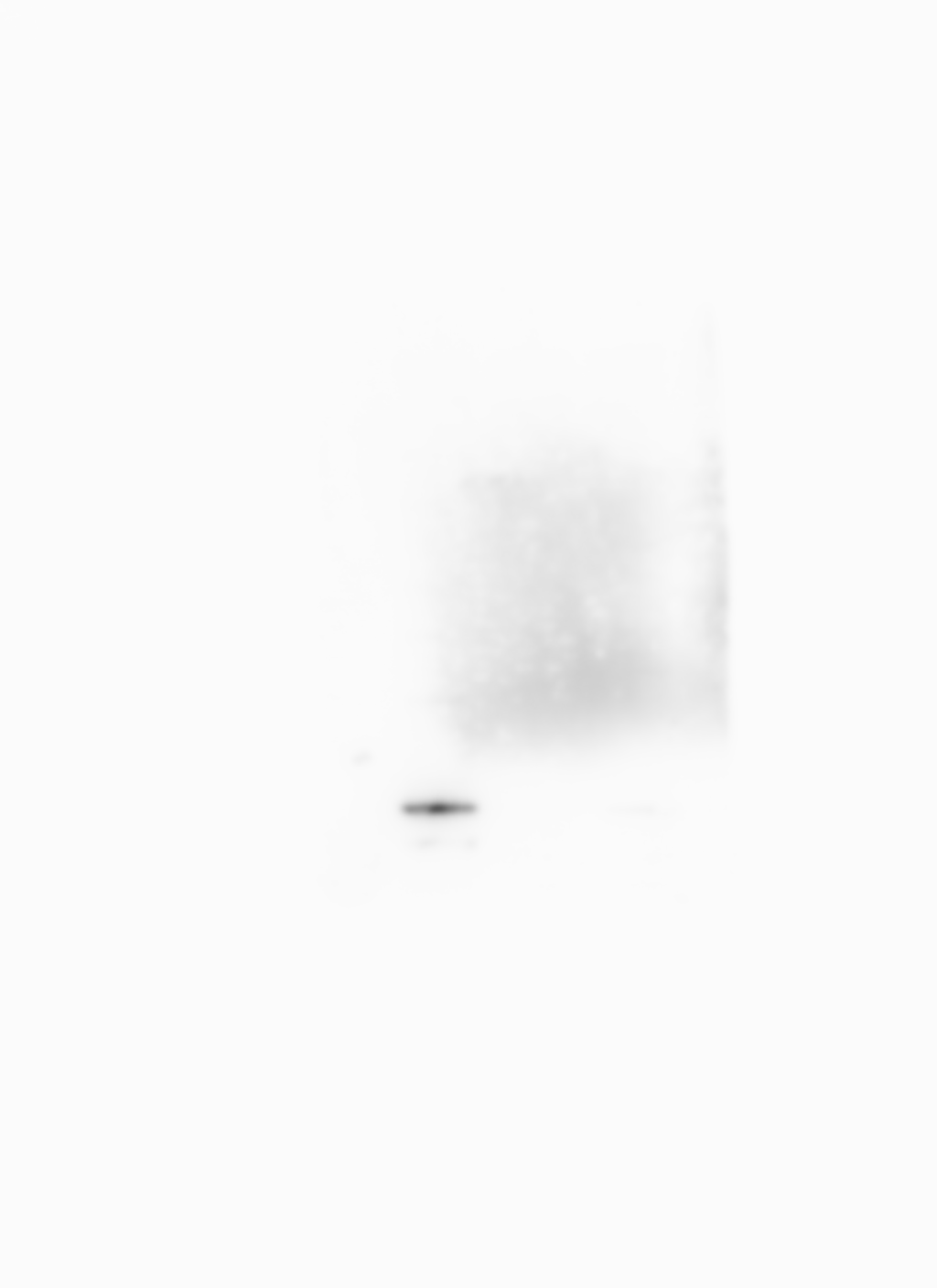

Supplement: Figure 3—figure supplement 1—source data 2. [file elife-99172-fig3-figsupp1-data2.zip › Figure 3-figure supplement 1A-Source Data2/CD81 of CD9-CD81 KOs AfterExperiments 2021.02.05_12.02.55_Ch.tif]

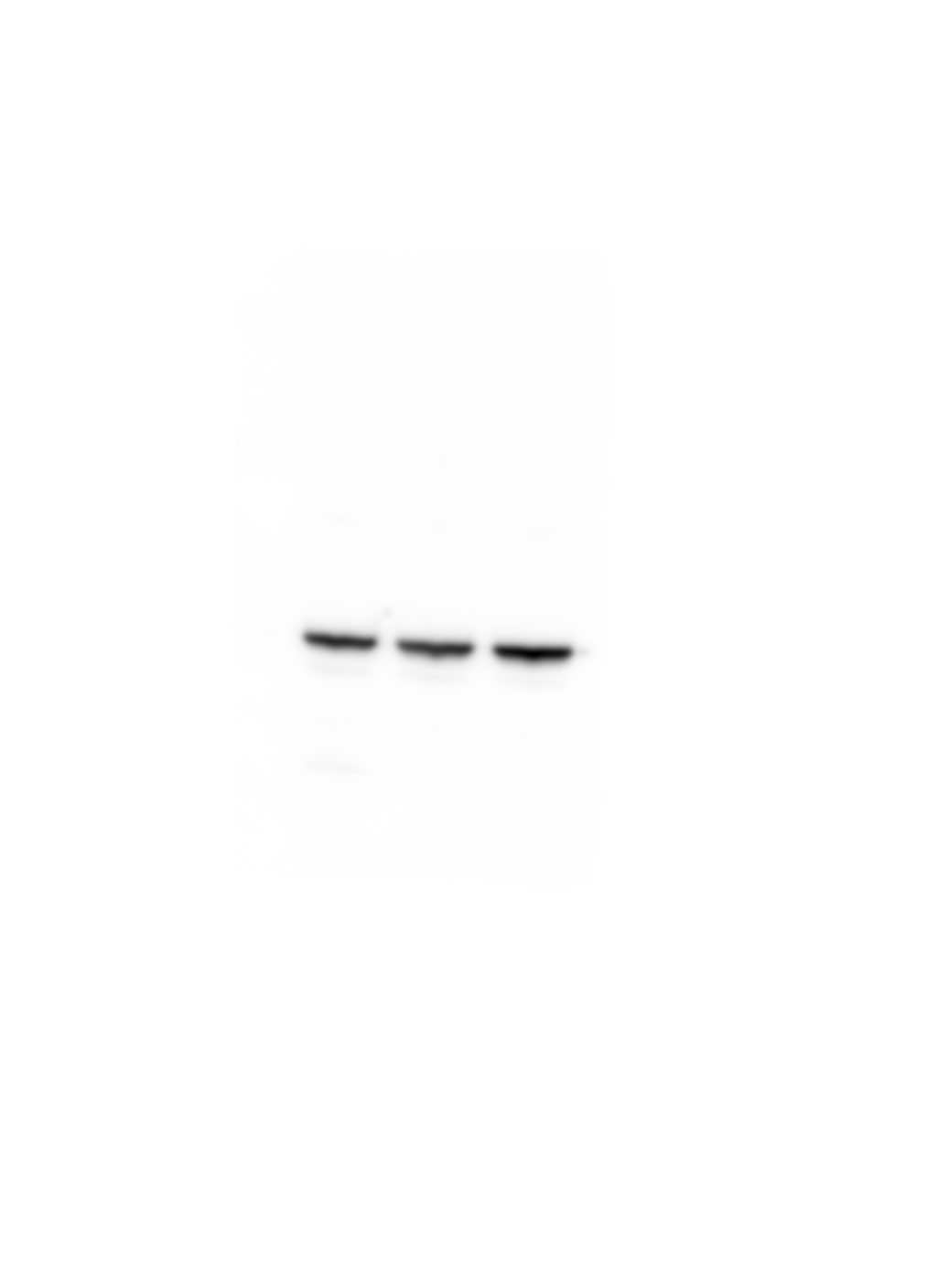

Supplement: Figure 3—figure supplement 1—source data 2. [file elife-99172-fig3-figsupp1-data2.zip › Figure 3-figure supplement 1A-Source Data2/GAPDH 1 of CD9-CD81 KOs AfterExperiments 2021.02.08_15.41.22_Ch.tif]

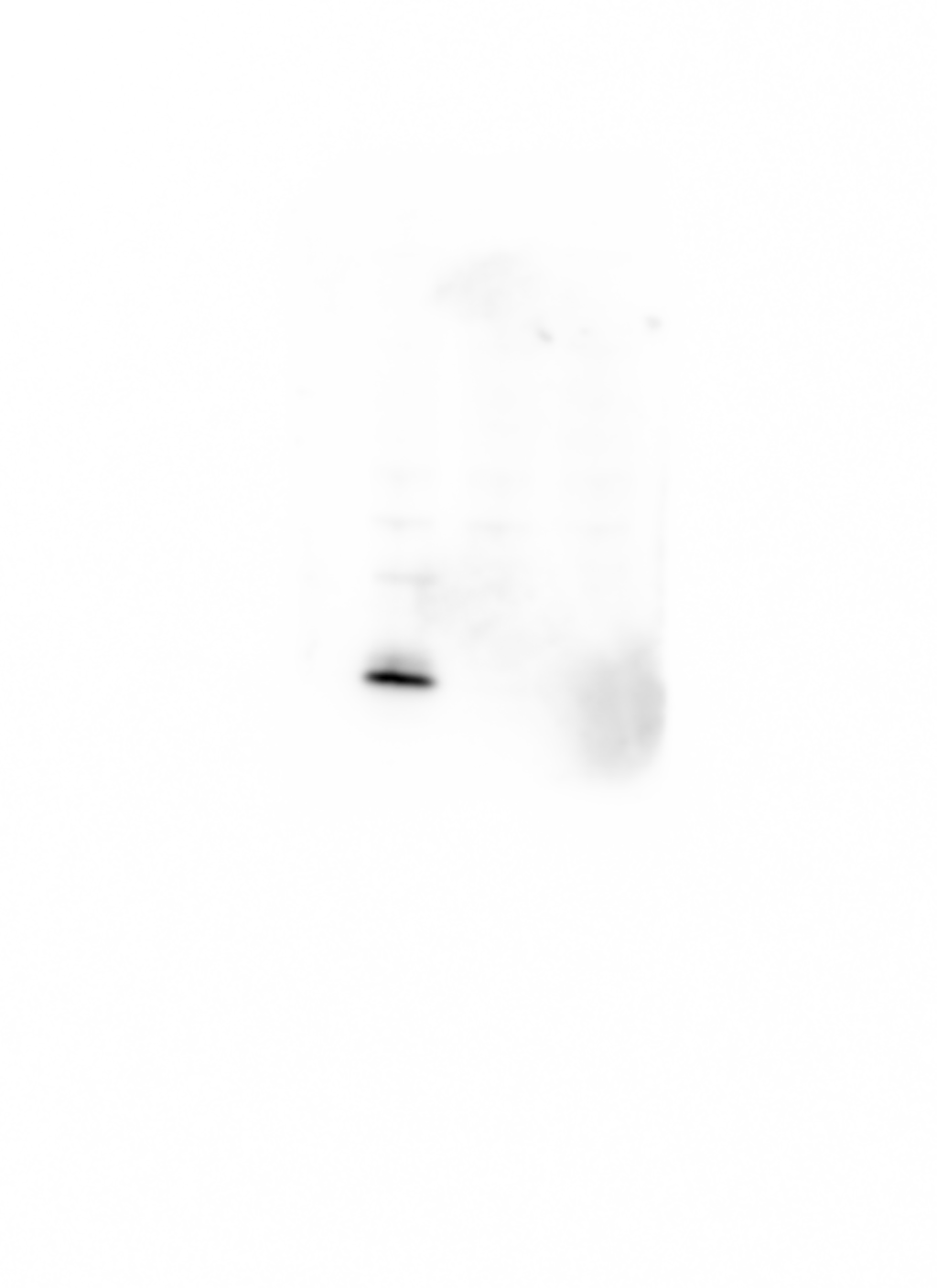

Supplement: Figure 3—figure supplement 1—source data 2. [file elife-99172-fig3-figsupp1-data2.zip › Figure 3-figure supplement 1A-Source Data2/CD9 of CD9-CD81 KOs AfterExperiments 2021.02.05_11.58.51_Ch.tif]

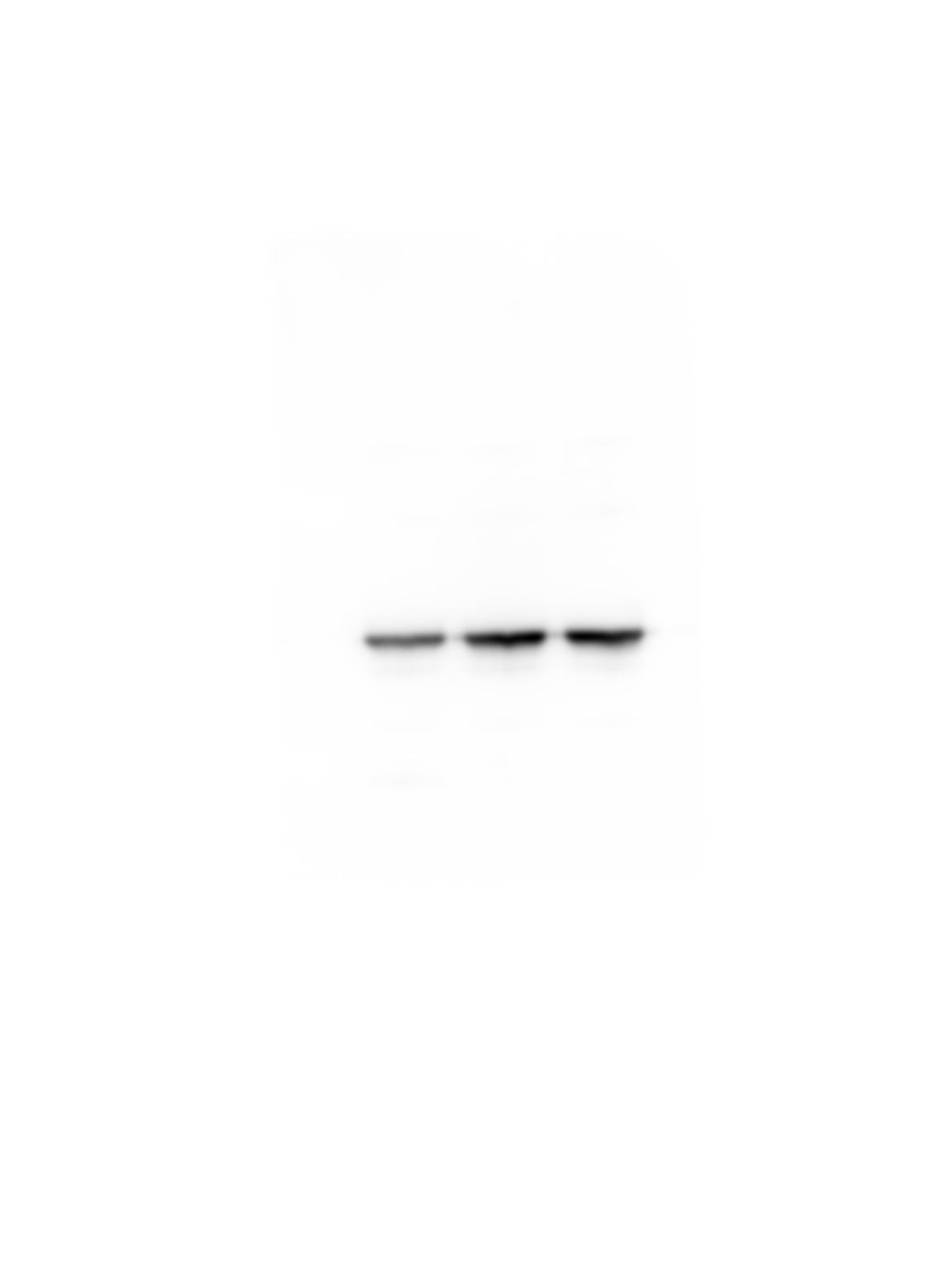

Supplement: Figure 3—figure supplement 1—source data 2. [file elife-99172-fig3-figsupp1-data2.zip › Figure 3-figure supplement 1A-Source Data2/GAPDH 2 of CD9-CD81 KOs AfterExperiments 2021.02.08_15.44.30_Ch.tif]

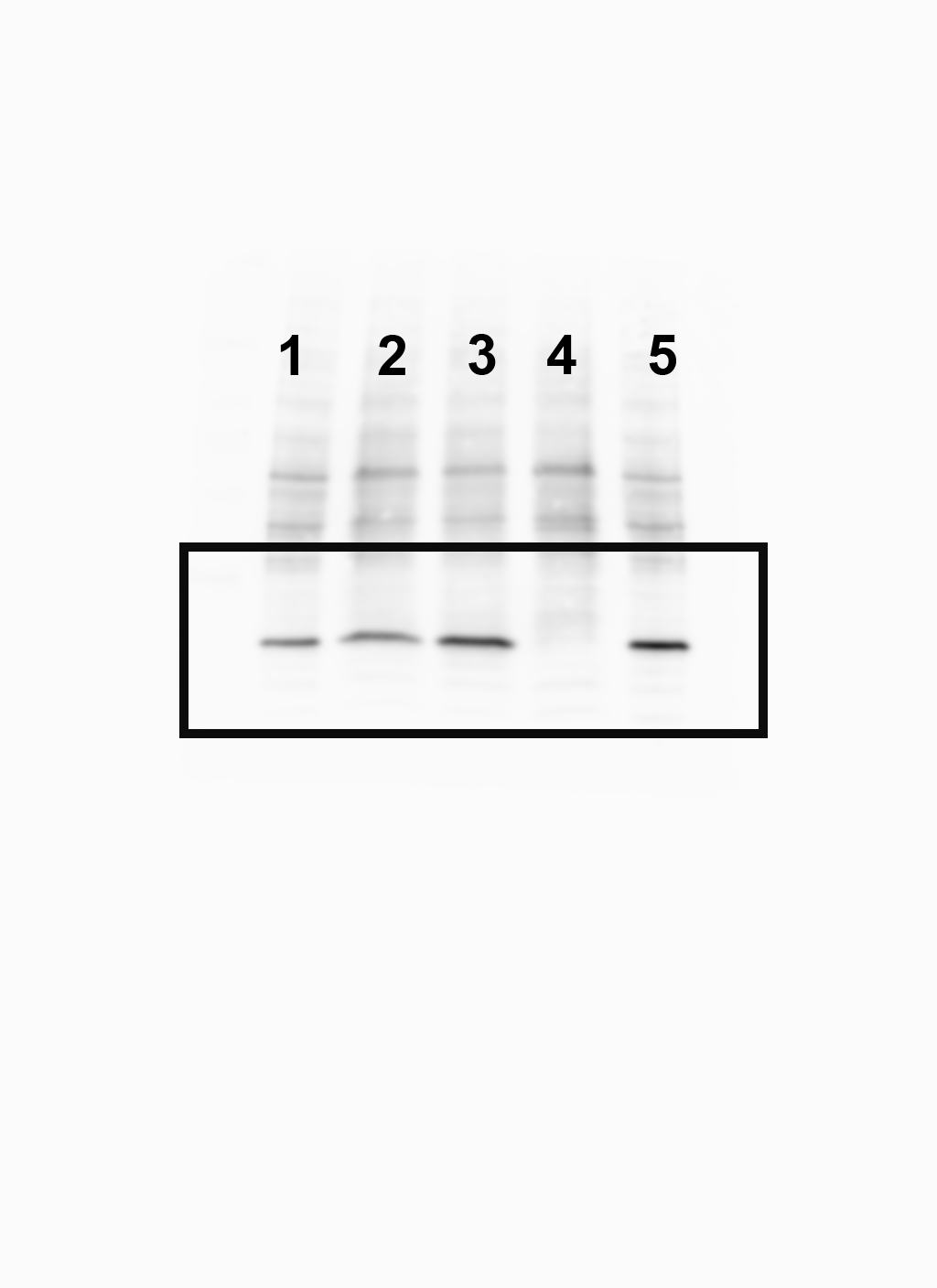

Supplement: Figure 3—figure supplement 1—source data 3. [file elife-99172-fig3-figsupp1-data3.zip › Figure 3-figure supplement 1C-Source Data 1/cd81 of cd9-81oe+cd9-81ko EXP2 2022.06.10_16.11.26_Chlabelled.tif]

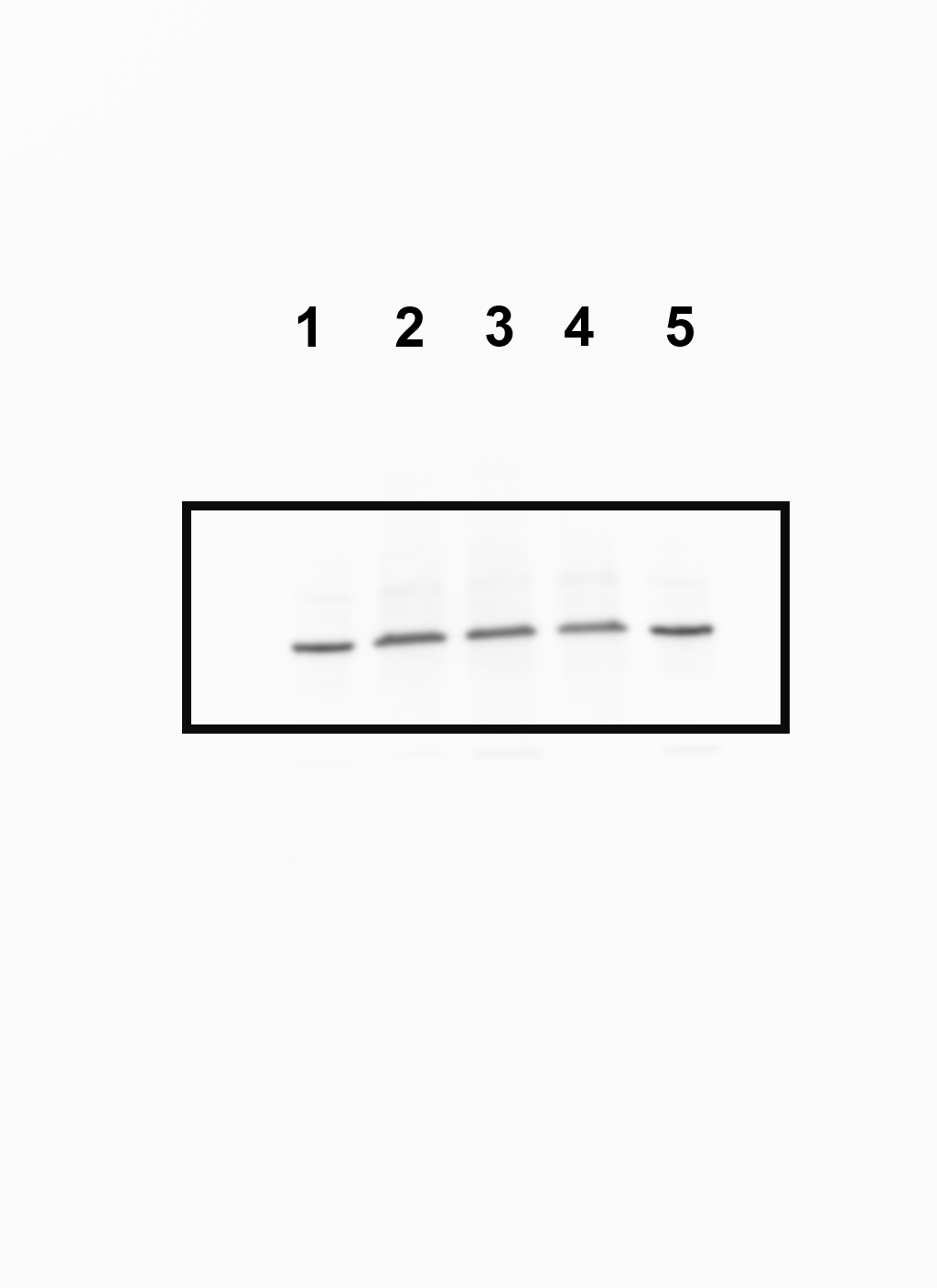

Supplement: Figure 3—figure supplement 1—source data 3. [file elife-99172-fig3-figsupp1-data3.zip › Figure 3-figure supplement 1C-Source Data 1/actin cd81 of cd9-81oe+cd9-81ko EXP2 2022.06.13_11.22.53_Chlabelled.tif]

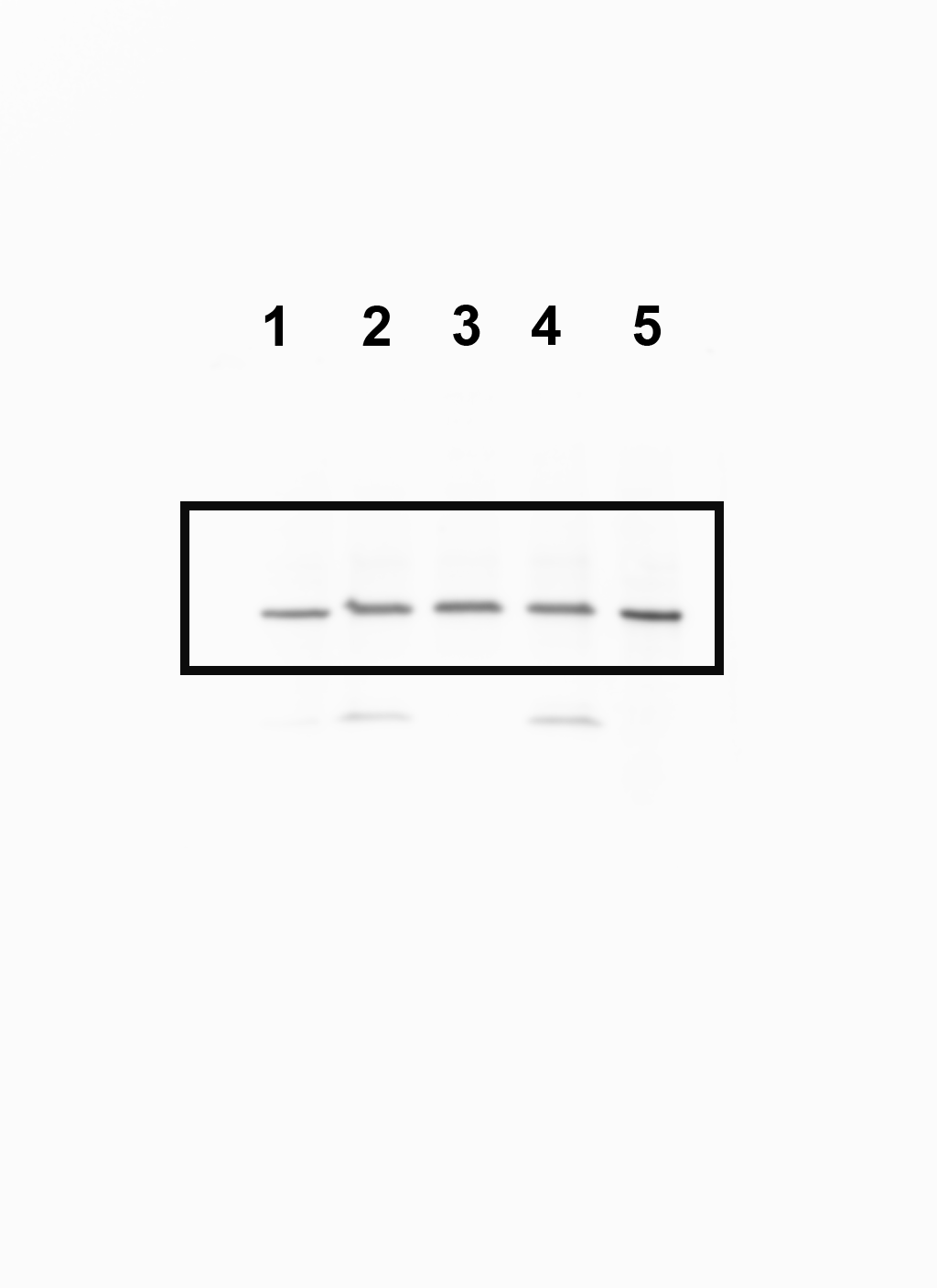

Supplement: Figure 3—figure supplement 1—source data 3. [file elife-99172-fig3-figsupp1-data3.zip › Figure 3-figure supplement 1C-Source Data 1/actin cd9 of cd9-81oe+cd9-81ko EXP2 2022.06.13_11.18.49_Chlabelled.tif]

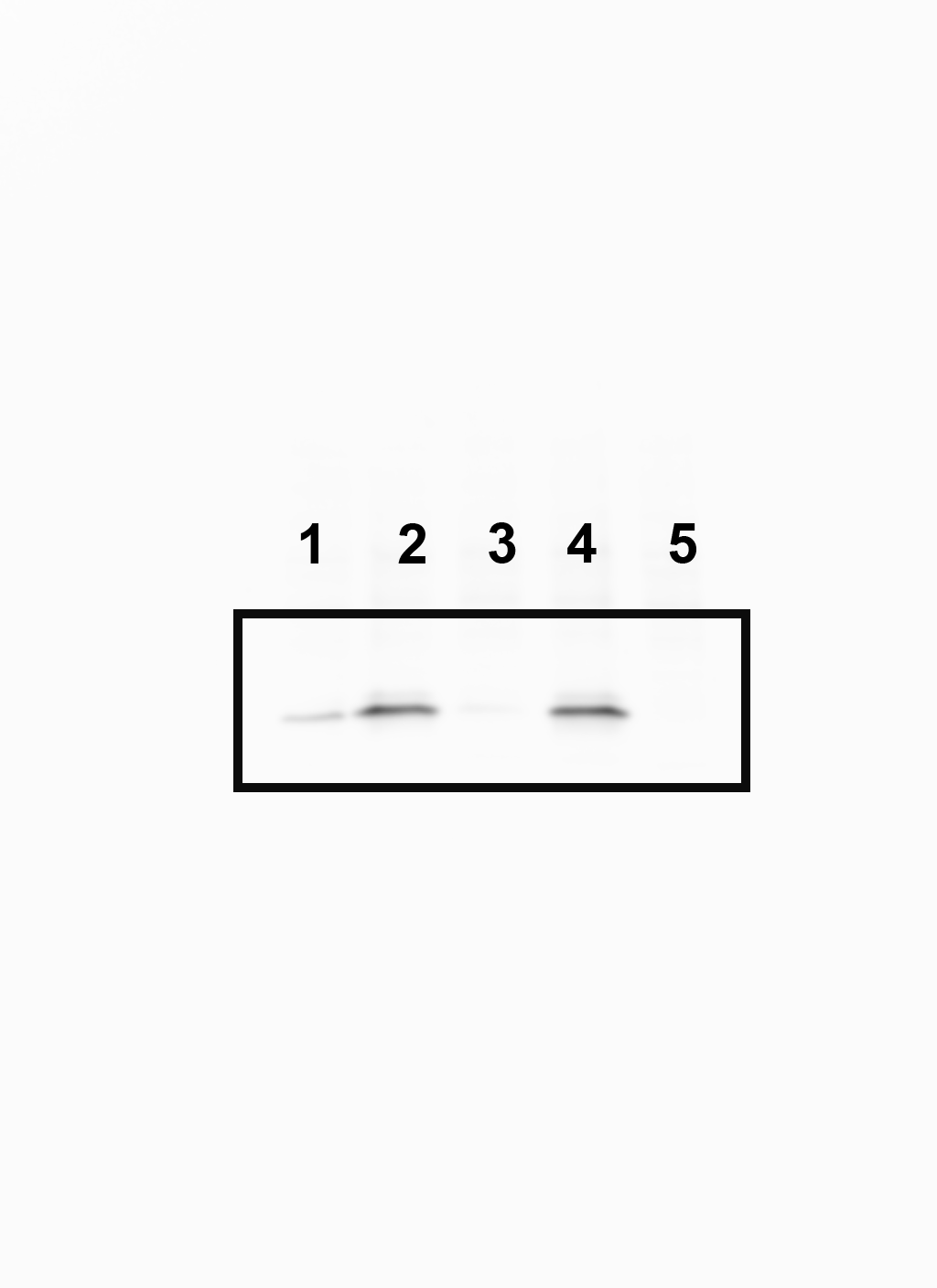

Supplement: Figure 3—figure supplement 1—source data 3. [file elife-99172-fig3-figsupp1-data3.zip › Figure 3-figure supplement 1C-Source Data 1/cd9 of cd9-81oe+cd9-81ko EXP2 2022.06.10_16.06.57_Chlabelled.tif]

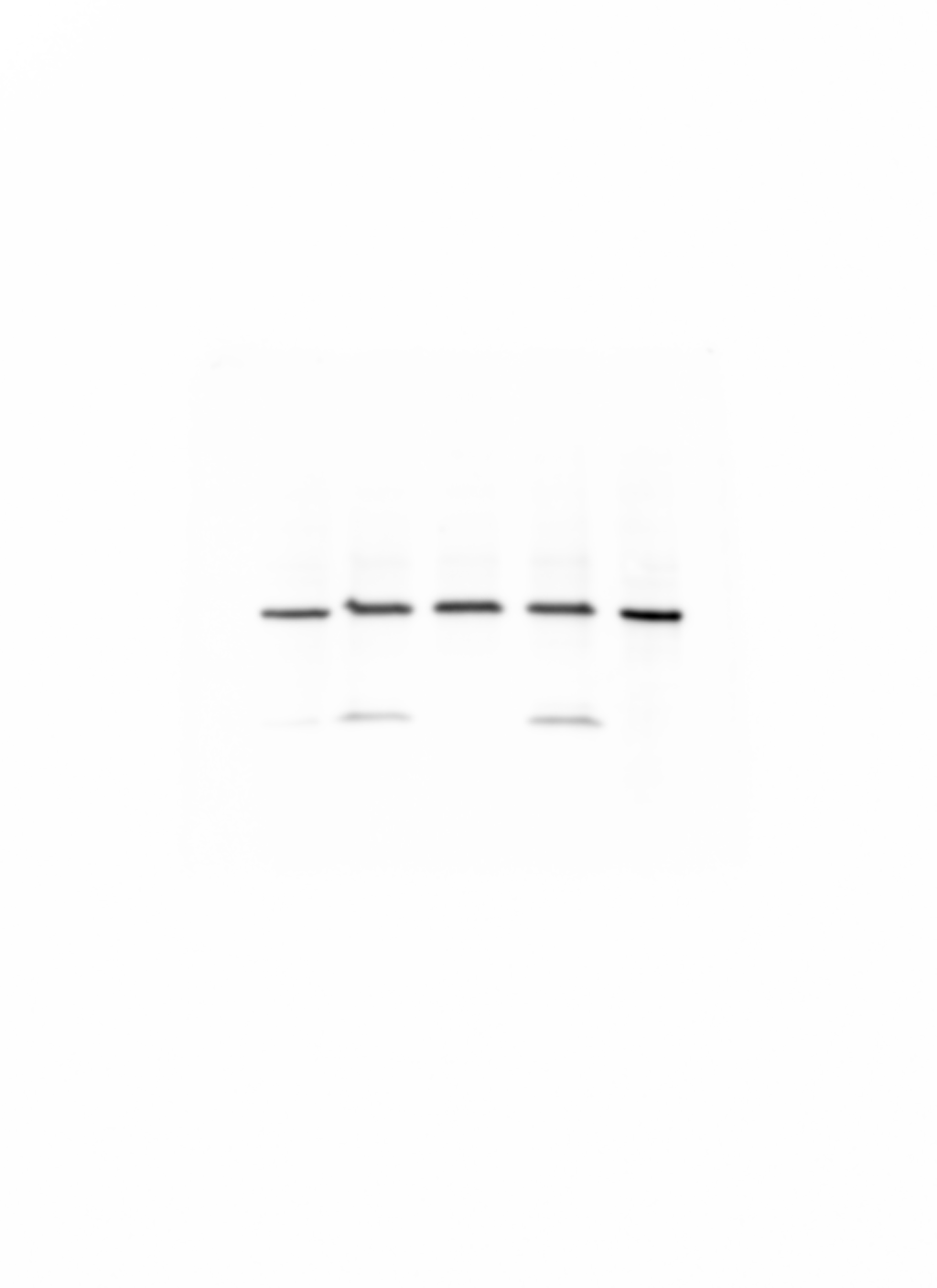

Supplement: Figure 3—figure supplement 1—source data 4. [file elife-99172-fig3-figsupp1-data4.zip › Figure 3-figure supplement 1C-Source Data2/actin cd9 of cd9-81oe+cd9-81ko EXP2 2022.06.13_11.18.49_Ch.tif]

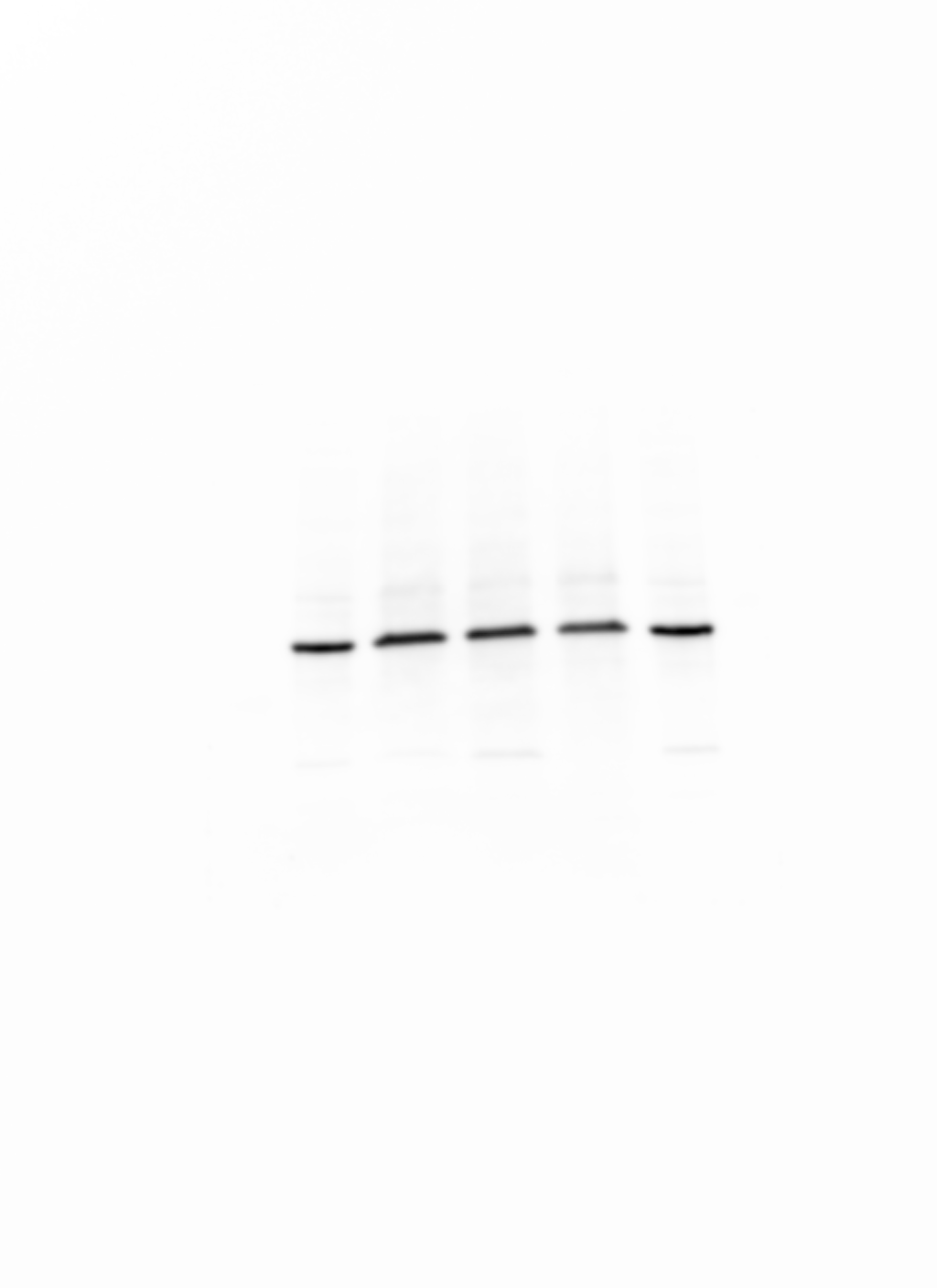

Supplement: Figure 3—figure supplement 1—source data 4. [file elife-99172-fig3-figsupp1-data4.zip › Figure 3-figure supplement 1C-Source Data2/actin cd81 of cd9-81oe+cd9-81ko EXP2 2022.06.13_11.22.53_Ch.tif]

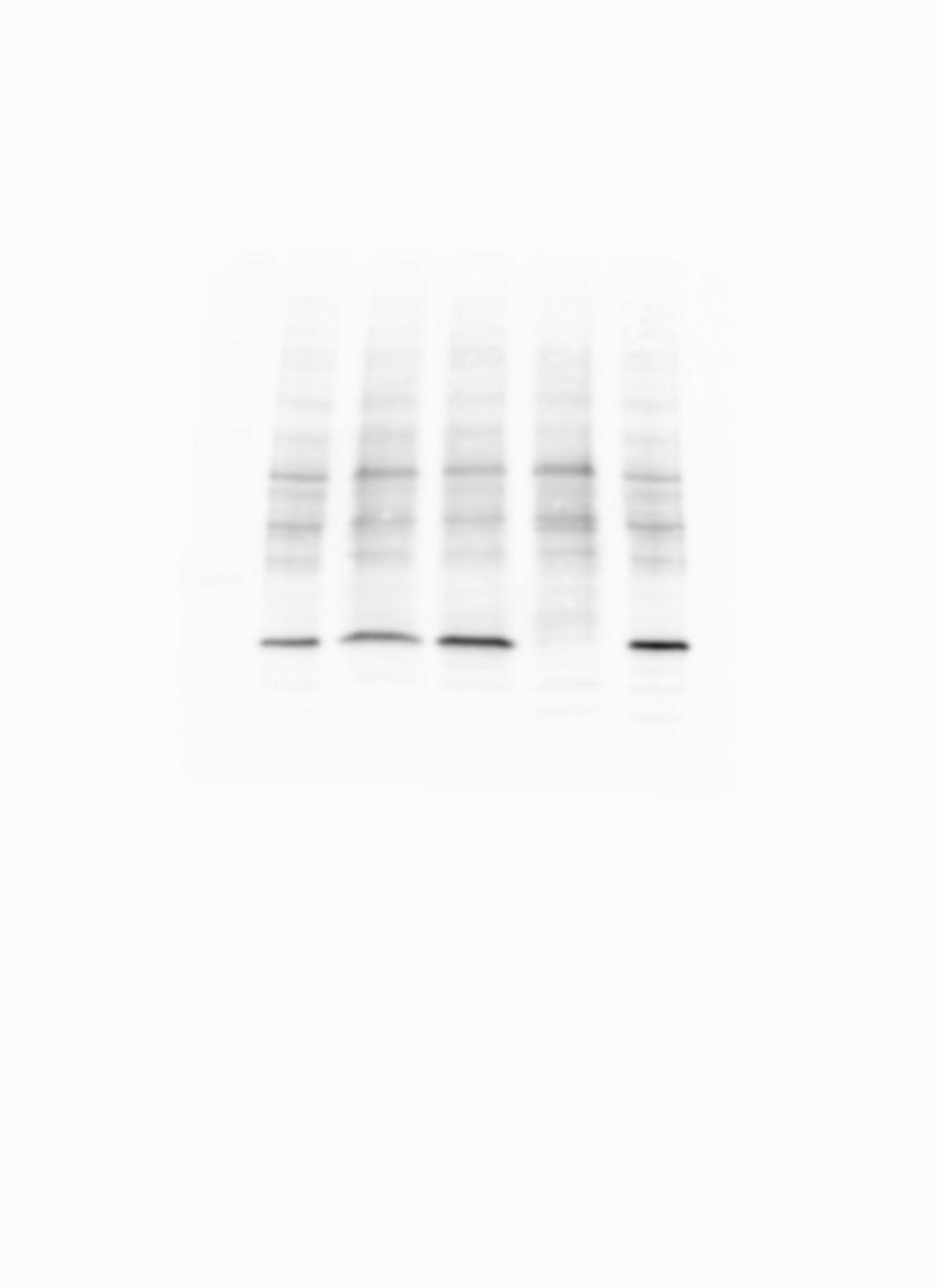

Supplement: Figure 3—figure supplement 1—source data 4. [file elife-99172-fig3-figsupp1-data4.zip › Figure 3-figure supplement 1C-Source Data2/cd81 of cd9-81oe+cd9-81ko EXP2 2022.06.10_16.11.26_Ch.tif]

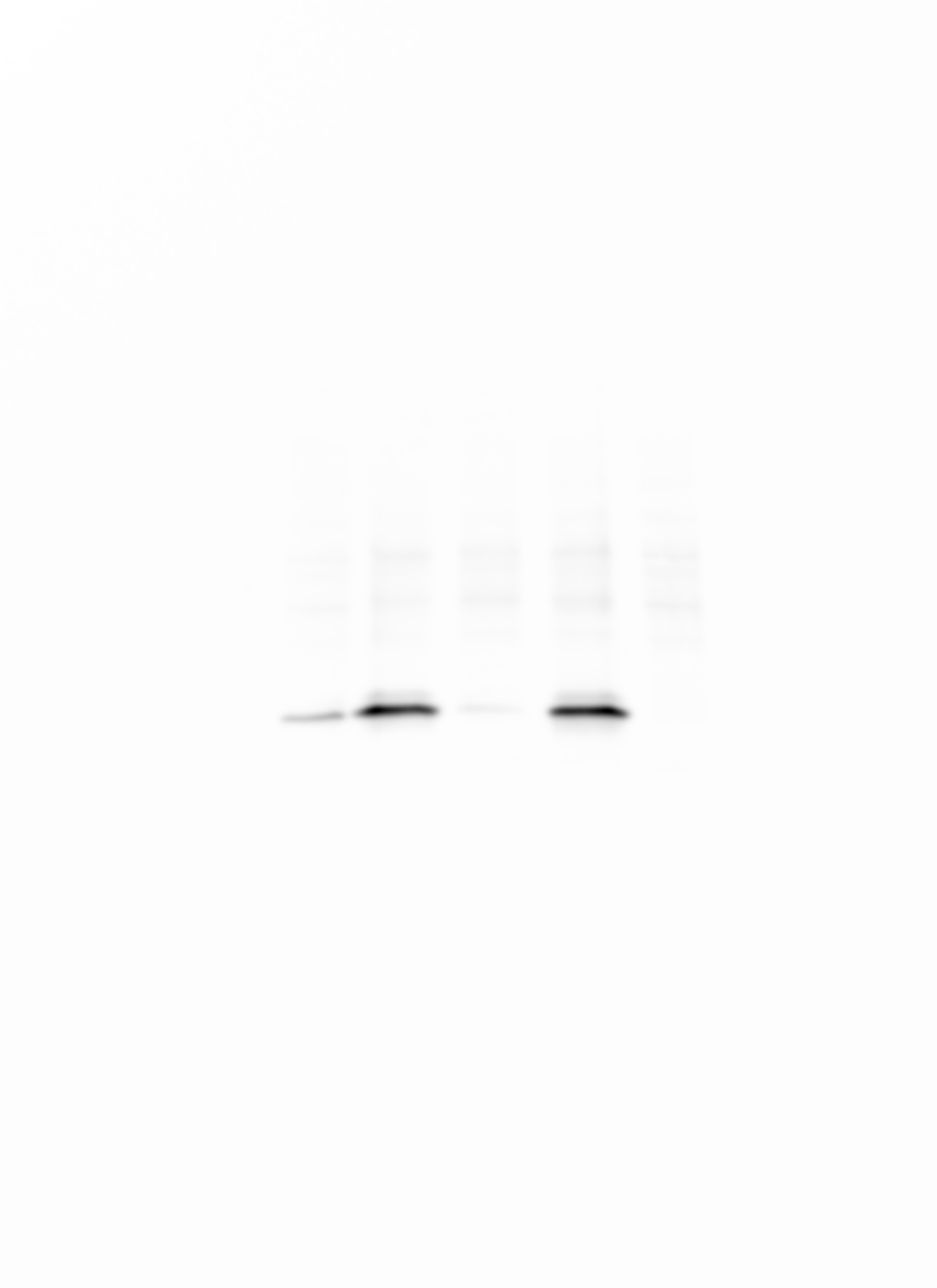

Supplement: Figure 3—figure supplement 1—source data 4. [file elife-99172-fig3-figsupp1-data4.zip › Figure 3-figure supplement 1C-Source Data2/cd9 of cd9-81oe+cd9-81ko EXP2 2022.06.10_16.06.57_Ch.tif]
